# A comprehensive survey of non-canonical splice sites in the human transcriptome

**DOI:** 10.1093/nar/gku744

**Published:** 2014-08-14

**Authors:** Guillermo E. Parada, Roberto Munita, Cledi A. Cerda, Katia Gysling

**Affiliations:** Nucleus Millennium in Stress and Addiction, Department of Cellular and Molecular Biology, Faculty of Biological Sciences, Pontificia Universidad Católica de Chile, Alameda 340, Santiago, Chile

## Abstract

We uncovered the diversity of non-canonical splice sites at the human transcriptome using deep transcriptome profiling. We mapped a total of 3.7 billion human RNA-seq reads and developed a set of stringent filters to avoid false non-canonical splice site detections. We identified 184 splice sites with non-canonical dinucleotides and U2/U12-like consensus sequences. We selected 10 of the herein identified U2/U12-like non-canonical splice site events and successfully validated 9 of them via reverse transcriptase-polymerase chain reaction and Sanger sequencing. Analyses of the 184 U2/U12-like non-canonical splice sites indicate that 51% of them are not annotated in GENCODE. In addition, 28% of them are conserved in mouse and 76% are involved in alternative splicing events, some of them with tissue-specific alternative splicing patterns. Interestingly, our analysis identified some U2/U12-like non-canonical splice sites that are converted into canonical splice sites by RNA A-to-I editing. Moreover, the U2/U12-like non-canonical splice sites have a differential distribution of splicing regulatory sequences, which may contribute to their recognition and regulation. Our analysis provides a high-confidence group of U2/U12-like non-canonical splice sites, which exhibit distinctive features among the total human splice sites.

## INTRODUCTION

Most genes in higher eukaryotes are interrupted by non-coding sequences, called introns, which are precisely excised from pre-mRNAs during splicing. Nuclear pre-mRNA introns are processed by the spliceosome, a complex macromolecular machine composed of five small nuclear RNAs and numerous proteins (1,2).

Proper intron recognition and removal rely on consensus sequences located at the intron/exon boundaries. Dinucleotide sequences at these boundaries have been found to be strongly conserved and relevant for proper splicing (3–5). Nearly all introns belong to the so-called U2-type, which are spliced by the major spliceosome and are flanked by GT–AG splice site dinucleotides. The most frequent exception to this rule are the U2-type GC–AG splice sites, comprising ∼0.9% of human splice sites (6). On the other hand, about 0.4% of the human splice sites belong to the U12-type. These introns are processed by the minor spliceosome and even though they were first described to have AT–AC dinucleotides at the intron/exon boundaries, the vast majority of them contain GT–AG sites (7). Indeed, the AT–AC sites comprise only ∼0.09% of the splice sites (6).

Despite the disruptive splicing effects that have mutations of splice site dinucleotides (3–5), introns with non-canonical splice sites (that is, with sequences other than GT–AG, GC–AG or AT–AC at the intron/exon boundaries) have been reported to be efficiently removed (6,8–12). These reported non-canonical splice sites have U2/U12-like splice site consensus sequences (U2/U12-like non-canonical splice sites). For instance, evolutionary conserved U2-like introns with GA–AG splice sites have been identified in FGFR genes (8,9) and a functional GT–TG splice site has been found in the GNAS gene (10,11). Although the first global analysis of splice sites in the human transcriptome, conducted 14 years ago, did not find confident evidence for non-canonical splice sites (13), most recent analyses based on expressed sequence tag (EST) sequences have reported U12-like non-canonical splice sites (6) and more examples of U2-like GT–TG introns (12).

The advent of high-throughput sequencing technologies has provided an unprecedented opportunity to explore the complexity of mammalian transcriptomes (14). For instance, analyses of RNA-seq data have resulted in the discovery of thousands of new splice sites and alternative splicing events in the human transcriptome (15–17). However, the high resolution power of high-throughput sequencing has not been used to generate a non-canonical splice site catalog on the human transcriptome.

To make a comprehensive analysis of non-canonical splice sites present in the human transcriptome, we have processed nearly 3.7 billion RNA-seq reads from 16 human tissues and a lymphoblastoid human cell line (GM12878). Our systematic analysis provides a list of high-confidence non-canonical splice sites and an insight into their characteristic features. Our comprehensive identification of non-canonical splice sites will improve the human transcriptome annotation. Further understanding of the mechanism underlying the recognition and processing of non-canonical splice sites could expand our knowledge of the splicing process. We provide the full annotation and quantification of the entire list of high-confidence canonical and non-canonical splice junctions for each analyzed human tissue (available as a UCSC Hub at http://54.214.245.35/Tracks/Splicing/hub.txt).

## MATERIALS AND METHODS

### Processing of RNA-seq data

We used the used RNA-seq data of GM12878 cell line provided by ENCODE project (18) and RNA-seq data of a mixture of 16 human tissues generated by Illumina Body Map 2.0 project (for additional information see Supplementary Data). The reads were processed in order to remove the adapters and low-quality sequences (PHRED score ≤ 10) with FASTX toolkit (http://hannonlab.cshl.edu/fastx_toolkit/index.html) and sickle (https://github.com/najoshi/sickle) for trimming of paired-end reads. After this, only the reads ≥50 nt long were kept. RNA-seq reads of GM12878 provided from the ENCODE project (18) (Supplementary Data) were aligned to the diploid genome of GM12878 (19) and the RNA-seq of the 16 human tissues mixture to the reference genome (hg19) using MapSplice (20). MapSplice was configured to detect canonical and non-canonical splice junctions with anchor length ≥8, a minimal intron size of 1 nt and allowing two mismatches 25 nt each.

### *Ab initio* detection of splice junctions

Splice junctions were extracted from unique gapped alignments that have an anchor ≥8 nt. To avoid false non-canonical introns derived from alignment errors, the read alignments that have non-canonical splice junctions were corroborated by BLAT (21) and were discarded if they had repetitive sequences present at Repbase (22) or low complexity sequences detected by DUST (23). A splice junction was considered present in a dataset of RNA-seq data if at least three different sequences supported the splice junction. GM12878 RNA-seq data were aligned to their paternal and maternal genome (diploid genome). We only considered those splice junctions that were in at least three coincident alignments in maternal and paternal GM12878 genomes.

We extracted splice junctions from cDNA and EST alignments provided by UCSC Genome Browser database (24). We removed cDNA/EST sequences that were repetitive or had low complexity (using Repbase and DUST). Splice junctions were extracted from unique gapped alignments that had an anchor ≥15 without mismatches. The splice junctions that were supported by three or more cDNA/EST sequences were considered as present in cDNA/EST alignments.

As the alignments to the reference genome can span over indels and single nucleotide polymorphisms (SNPs), false non-canonical splice junctions could be detected. For this reason, we discarded the gapped alignments to the reference genome which had non-canonical splice junctions that spanned over indels or SNPs reported on SNPdb135.

### Library-guided detection of splice junctions

**Figure 1.**
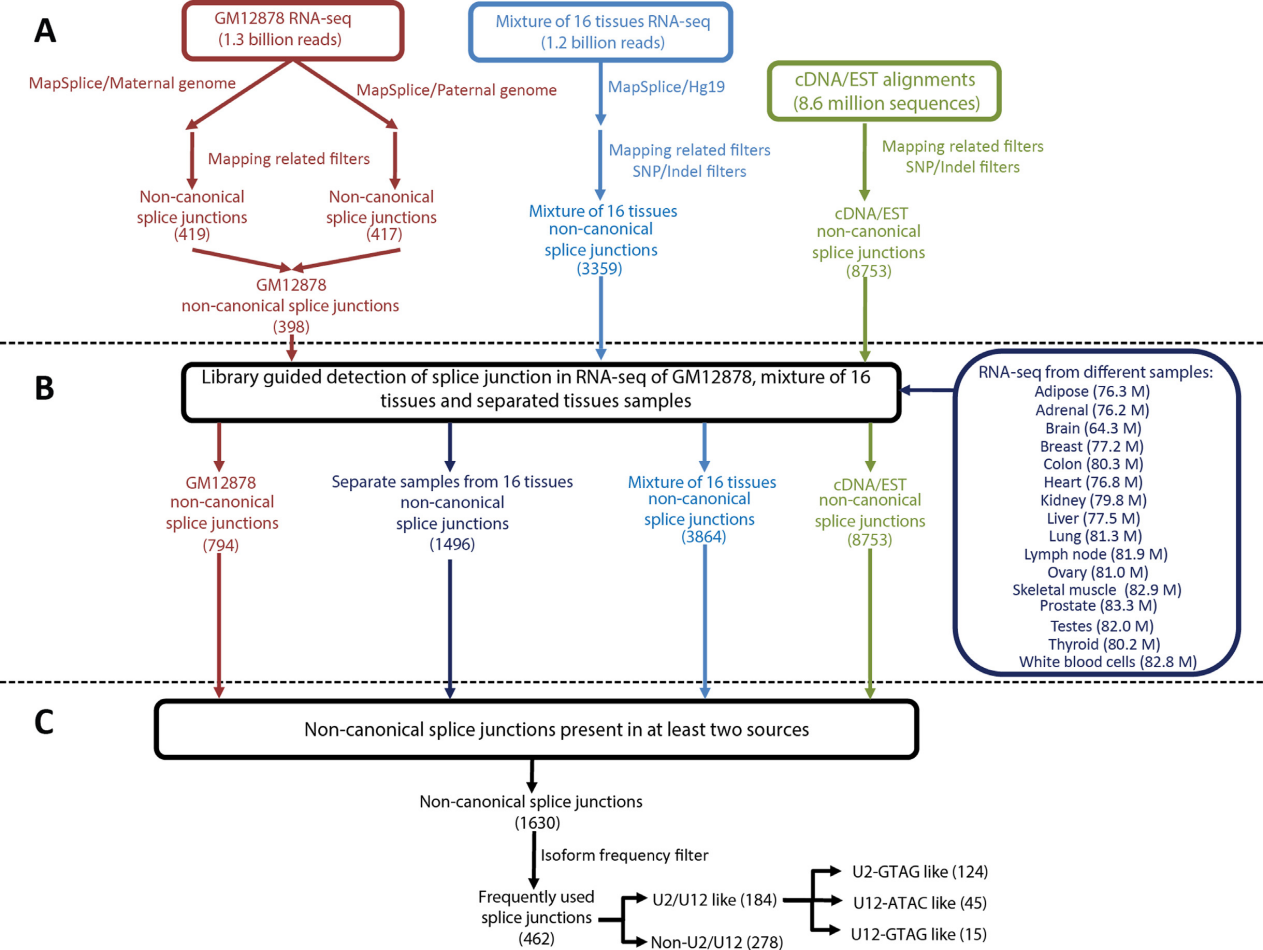
An overview of the workflow used for the search of non-canonical splice junctions. (**A**) *Ab initio* detection of non-canonical splice junctions. RNA-seq data of GM12878 were aligned to their diploid personalized genome and RNA-seq data from a mixture of 16 human tissues were aligned to the human reference genome (hg19) using MapSplice. All alignments of RNA-seq and cDNA/EST data were pre-processed in order to generate an initial library of splice junctions. Additional SNP/indel filters were applied to tissue RNA-seq and cDNA/EST alignment. (**B**) All the RNA-seq data were re-aligned to the library of splice junctions. Additional RNA-seq data from individual tissues were also directly aligned to the library. (**C**) A total of 1630 non-canonical introns were present in at least two sources of data. From these, 462 non-canonical splice junctions were detected in at least a coverage ratio of 1:20 compared with their most abundant splice variant.

All the splice junctions identified in RNA-seq and cDNA/EST alignments were used to create a library of splice junctions (Figure 1A). We mapped the previously used RNA-seq data, in addition to tissue-specific RNA-seq data from 16 human tissues, to the library of splice junctions using Bowtie (25), configured to align with a SOAP-like ‘-v 2’ mode allowing two mismatches with a seed length of 50 nt (Figure 1B). We checked that the new splice junction mapping reads found did not map to other genomic location and did not have repetitive or low complexity sequences. Moreover, to ensure the reproducibility of the splice junction detection, we selected the splice junctions that were present in at least two different data sources (tissues extracted from the same individual were considered as one data source) (Figure 1C).

### Non-canonical splice junction processing

Direct repeat sequences located at the splice junctions generate alignment ambiguity and multiple putative non-canonical splice sites. This phenomenon can lead to false non-canonical splice site annotation (13). For this reason, when a non-canonical splice junction was flanked by direct repeats, we checked that it did not have ambiguous canonical splice sites (13). Moreover, based on the position weight matrices (PWMs) reported for canonical human splice junctions (6), we selected the alignment that yields the non-canonical splice junction with the best fit into the canonical PWMs.

Based on the canonical splice junction PWMs, we classified the non-canonical introns as U2/U12-like or non-U2/U12. To do this, we assigned scores based on the PWMs to a population of splice junctions with scrambled sequences. We took the 95th percentile of the scrambled splice junction scores as the U2/U12 threshold score to classify non-canonical splice junction as U2/U12-like. If a non-canonical splice junction had a score higher than 70.00, it was classified directly as U2/U12-like. The non-canonical splice junctions that have scores between 70.00 and the threshold were classified as U2/U12-like only if they shared a splice site with a canonical splice junction. Finally, if the non-canonical splice junction had a score below the threshold score or its intron was smaller than 80 nt, it was classified as non-U2/U12.

### Alternative splice site analysis

After computing all the 5′ and 3′ splice sites identified, we classified the alternative splice site events in exon skipping, exon inclusion, intron retention and 5′/3′ alternative splice sites. Based on the total RNA-seq coverage of each splice junction, we estimated non-canonical intron usage percentages (ψ). To select the non-canonical splice junctions that could have functional relevance, we filtered the alternative non-canonical splice junctions. We only continue working with the non-canonical splice junctions that were detected in at least a coverage ratio of 1:20 compared with the predominant splicing variant.

### Splice junction annotation as a UCSC Genome Browser track

We annotated the splice junctions found as a UCSC Genome Browser track (available as a UCSC Hub at http://54.214.245.35/Tracks/Splicing/hub.txt). The splice junctions are represented as an intron flanked by two exons of 8 nt. The ID of each splice junction encodes its splice site dinucleotides and read coverage. U2/U12-like introns are in a green scale, where lighter green colors indicate higher similitude with U2/U12 splice junctions. Non-U2/U12 introns are in a red scale, where lighter colors indicate higher differences with U2/U12 splice junctions. Moreover, all detected non-canonical splice sites are reported with their 0-based genomic coordinates (Supplementary Data).

### Conservational analysis of the splice junctions

We extracted the splice junctions from mouse cDNA/EST alignments provided by the UCSC database (24) using the same processing protocol previously described for human cDNA/EST alignments. To compare the mouse and human splice junctions, we use LiftOver utility (24) to translate mouse genome coordinates to human genome coordinates. The splice junction was considered conserved if it had the same coordinates and splice site dinucleotides.

### Analysis of splicing regulatory sequence distribution

We map the hexamers associated with splicing regulatory elements (SREs) that were identified by fluorescence-activated screening (FAS) (26–29) to the human genome. The SRE hexamers were grouped by EIEs (ESE + ISS) and IIEs (ISE + ESS) (30). We took 5′/3′ exonic and intronic windows of 100 nt long for all the splice junctions identified. We compared the density of SRE hexamers (number of SRE standardized by length of the genome area analyzed) in the different windows between canonical and non-canonical splice junctions. The significance of the differences found was assessed by Pearson Chi-squared test.

### Experimental validation of non-canonical splice junctions by RT-PCR/Sanger sequencing

We used reverse transcriptase-polymerase chain reaction (RT-PCR) to validate 10 non-canonical U2/12-like and 4 non-U2/U12 splice junctions. We used Human Universal RNA samples (SABiosciences) and different RTs: MMLV (RevertAidFirst Strand cDNA Synthesis), AMV (Promega) or Transcriptor (Roche) to do the RT-PCR. The PCR amplification was carried out using a touchdown thermocycling program (31) during 35 cycles and products were analyzed by gel electrophoresis. The presence of the different isoforms detected were corroborated using Sanger sequencing of the RT-PCR products subcloned using the pGEM-T Easy Vector System (Promega). The primers for the RT-PCR are listed in Supplementary Table S1.

### Secondary RNA structure prediction

*In silico* prediction of secondary RNA structure associated with non-U2/U12 splice junction was performed using RNA fold web server (32). The visualization of the RNA structures was obtained using PseudoViewer3 (33) and edited with Adobe Illustrator.

## RESULTS

### Using public transcriptome data to find high-confidence splice junctions

We have done a comprehensive search of canonical and non-canonical splice sites at the human transcriptome using public RNA-seq data and cDNA/EST data. Mapping RNA-seq data coming from different genomic backgrounds to the human reference genome can lead to false non-canonical splice junction detection due to indels in the reference genome or SNPs at the splice sites; thus we used the transcriptomic and genomic data from the same cell line (GM12878). Both sources of sequences derive from the same genomic background and, therefore, with the same genomic variations. The GM12878 is a lymphoblastoid cell line that has been deeply sequenced with both parents (as a trio) and ensembled as diploid genome, with maternal and paternal allele specific genomic variations (19). Using MapSplice (20), we first mapped 1.3 billion of RNA-seq reads from GM12878 (provided by the ENCODE project (18)) to the diploid genome of GM12878 and obtained non-canonical splice junctions free of genome variation associated errors (Figure 1A). Then, to get a comprehensive landscape of non-canonical splice sites at the different human tissues, we also analyzed the alignments of 1.2 billion RNA-seq reads from a mixture of 16 human tissues (Illumina Body Map 2.0) to the human reference genome and cDNA/EST alignments, but we filter non-canonical splice sites that have SNPs or indels reported in SNPdb135 (34) (Figure 1A).

Although MapSplice has shown high performance to discover novel splice junctions, *ab initio* detection of splice junctions is a computationally challenging task prone to sensitivity problems (35–37). In order to increase the sensitivity to identify RNA-seq reads coming from splice junctions, we directly mapped a total of 3.7 billion human RNA-seq reads from GM12878 (1.3 billion), a mixture of 16 human tissues (1.2 billion) and RNA-seq from individual tissue samples (1.2 billion) to the splice junction library of all introns that we initially found (Figure 1B). By doing this, we increased the number of reads that align to non-canonical splice junctions. We found 1630 non-canonical splice sites present in at least two different sources of data (Supplementary Data). For the following analysis, we only considered the 462 non-canonical sites that were found to have at least a coverage ratio of 1:20 compared with their most abundant splice variant (Figure 1C). We identified 278 non-U2/U12 non-canonical splice sites (Figure 1C). Non-U2/U12 non-canonical splice sites were analyzed separately because they could be attributed to sequencing errors and reverse transcriptase template switching events.

### Diversity of U2/U12-like non-canonical splice sites

We found 184 high-confidence non-canonical introns that have U2/U12-like splicing sequences (Figure 1C, Table 1 and Supplementary Data), of which 51% are not annotated in GENCODE v17. Thus, this represents a group of introns rich in novel and unexplored splice junctions present in the human transcriptome.

**Table 1.**
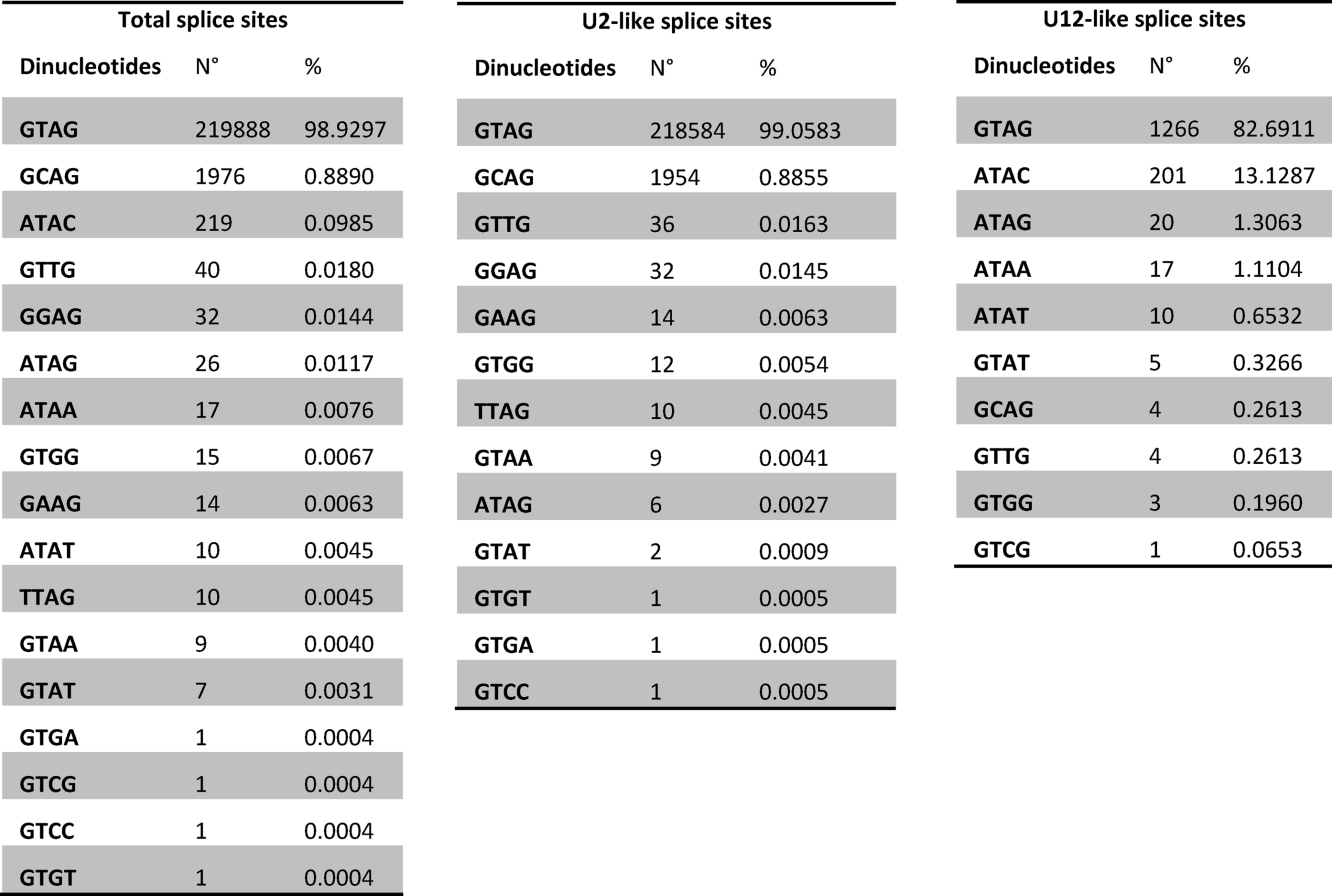
Diversity of human splice sites

We validated 9 of 10 selected U2/U12-like non-canonical introns by RT-PCR using a human RNA sample representative of many major human tissues (Supplementary Figures S1–S9). The sequences obtained by Sanger method were aligned to the human reference genome showing the same non-canonical introns identified herein.

### Evolutionary conservation of U2/U12-like non-canonical splice sites

**Figure 2.**
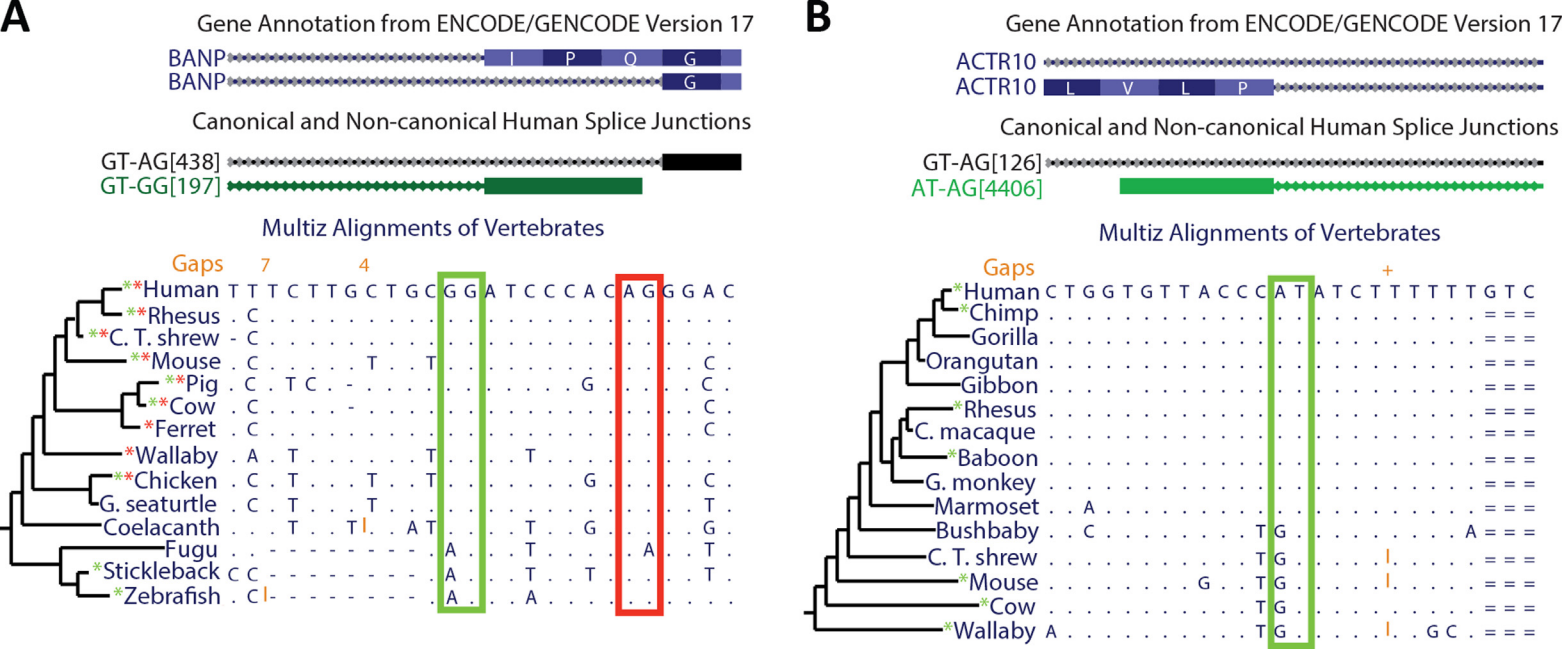
Non-canonical splice site conservation across different vertebrates. The UCSC Genome Browser images show two examples of non-canonical splice sites that are conserved in several vertebrates. Based on our results, we made an annotation of human splice junctions (middle track). The splice junctions' name (ID) indicates their dinucleotides and read coverage (for details see the Materials and Methods section). Genome alignments from different vertebrates are shown. Dots indicate conservation of the human nucleotides; red and green rectangles indicate the conservation of canonical and non-canonical splice sites respectively; and * indicates that the splice site is supported by cDNA/EST alignments of each vertebrate. (**A**) The BANP gene has an alternative GG-3′ non-canonical splice site that is conserved among most of the vertebrates and it was derived from an ancient canonical splice site. (**B**) The AT–AG non-canonical splice site of ACTR10 has been recently derived in evolution from a canonical splice site. This non-canonical splice site is exclusive of some primates.

To assess the evolutionary conservation of U2/U12-like non-canonical splice junctions, we verified their presence in mouse cDNA/EST alignments. We found that at least 28% of identified human non-canonical splice sites are conserved in mouse. Moreover, there are non-canonical splice sites conserved across large evolutionary distances. For example, the GT–GG splice sites of BANP gene show a transversal conservation among almost all vertebrates, but in fishes it is processed as GT–AG canonical splice sites, because the 3′ GG splice site is a 3′ AG in the fish genome (Figure 2A). In contrast, the AT–AG non-canonical splice sites present in ACTR10 gene are only conserved in some primates because the 5′ AT splice site is a 5′ GT in other mammalian genomes (Figure 2B).

### Most of U2/U12-like non-canonical splices are involved in alternative splicing

**Figure 3.**
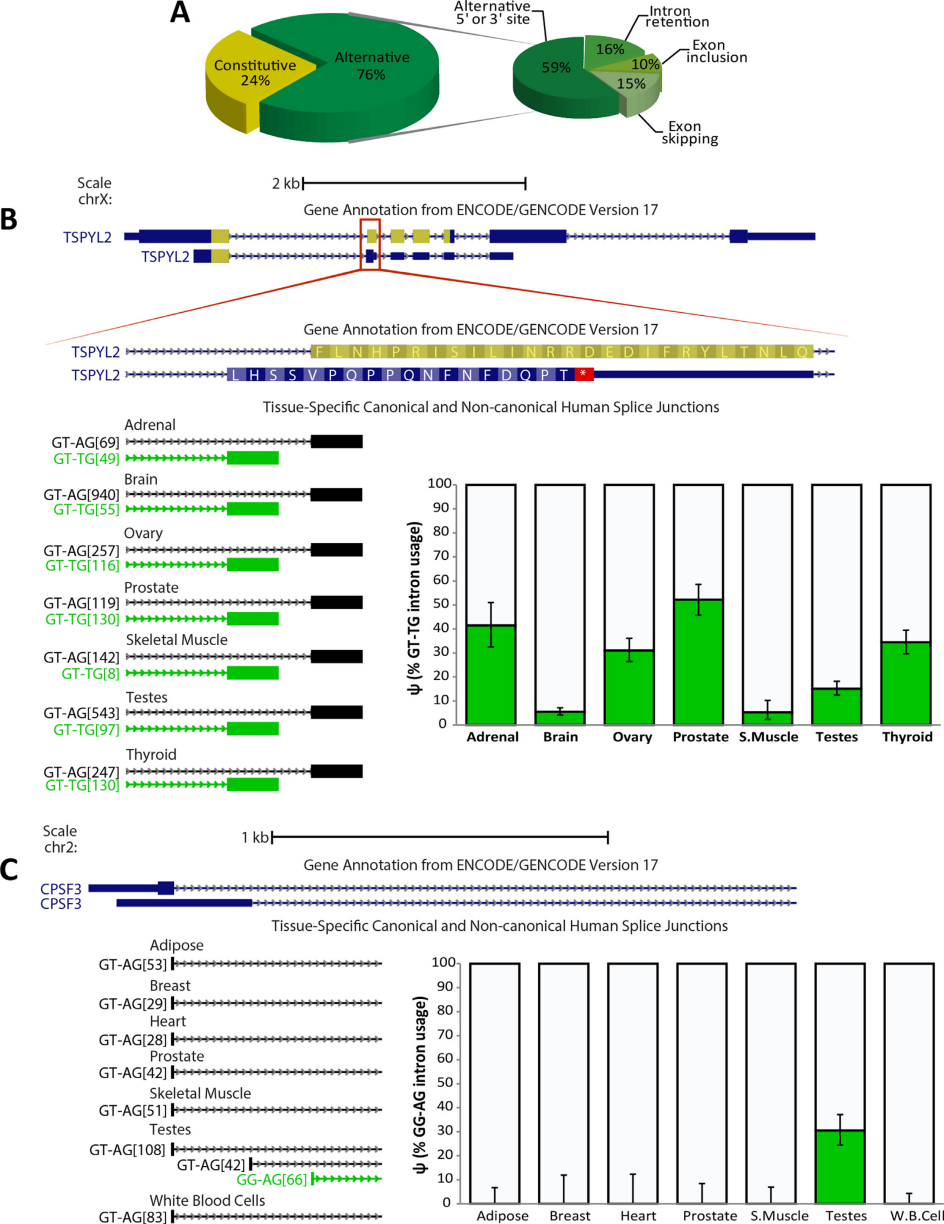
Non-canonical splice sites are highly involved in alternative splicing and some show tissue-specific alternative splicing patterns. (**A**) Participation of non-canonical splice sites in alternative splicing. (**B**) The use of a non-canonical splice site generates a frame shift and a premature termination codon that disrupts a NAP-like domain (highlighted in yellow) of the TSPYL2 protein. A tissue-specific pattern of 3′ alternative splice site selection is shown across seven human tissues. Coverage quantification is plotted, where error bars indicate the 95% binomial confidence interval. (**C**) CPSF3 gene has an alternative 5′-GG non-canonical splice site. Coverage quantification shows a testis-specific selection of the 5′-GG non-canonical splice site.

Our analysis shows that 76% of U2/U12-like non-canonical splice sites are involved in alternative splicing events, while 46% of canonical splice sites are involved in alternative splicing events. Moreover, we found that alternative 5′/3′ splice sites are the most abundant kind of alternative splicing events associated with U2/U12-like non-canonical splice sites (Figure 3A), consistent with previous reports (8,12).

### U2/U12-like non-canonical splice site involvement in tissue-specific alternative splicing

Some introns that have U2/U12-like non-canonical splice sites are alternatively spliced with tissue-specific patterns. One example is a GT–TG splice site located at the TSPYL2 gene. The TSYPL2 gene encodes a protein that is part of the CASK/TRB1/TSPYL2 transcriptional complex which modulates gene expression in response to neuronal synaptic activity, probably by using its nucleosome assembly protein-like (NAP-like) domain to modulate nucleosome assembly (38). The NAP-like domain is interrupted by a premature stop codon when an alternative TG 3′ splice site is used. In tissues like adrenal and prostate, the TG 3′ splice site is used in more than 40% of the transcripts, but, in contrast, in brain and skeletal muscle, the TG 3′ splice site is used in less than 10% of the transcripts (Figure 3B).

We found non-canonical splice sites that are highly tissue specific. For example, the CPSF3 gene, which encodes a key component of the cleavage and polyadenylation factor complex, has a GG–AG non-canonical splice site that is only used on testis transcripts (Figure 3C). This non-canonical splice site is currently not annotated in GENCODE v17, probably due to its high tissue-specific pattern.

### U2/U12-like non-canonical splice sites show differences in the abundance of splicing regulatory sequences

*Cis*-acting SREs are critical for splicing modulation (39). Several studies have identified exonic splicing enhancers (ESEs) (26), exonic splicing silencers (ESSs) (27), intronic splicing enhancers (ISEs) (28) and intronic splicing silencers (ISSs) (29) by FAS (40). To determine if there are different SRE patterns between canonical and non-canonical splice sites, we mapped hexamers enriched in the SRE sequences that were identified by FAS to the human reference genome and counted their occurrences around all identified splice sites.

**Figure 4.**
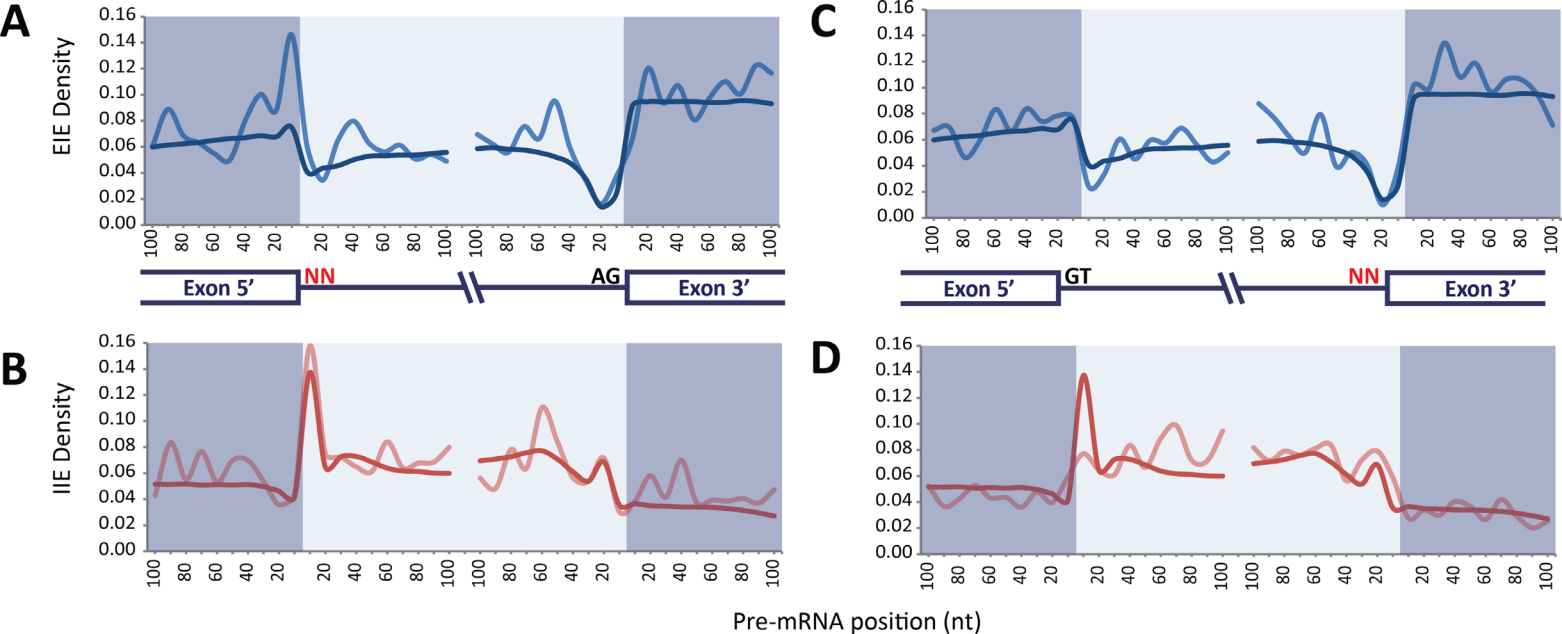
Non-canonical site junctions have distinctive distribution of EIE and IIE. (**A**) Positional density of EIE in canonical (blue) and 5′ non-canonical (pale blue) splice sites. (**B**) Positional density of IIE in canonical (red) and 5′ non-canonical (pale red) splice sites. (**C**) Positional density of EIE in canonical (blue) and 3′ non-canonical (pale blue) splice sites. (**D**) Positional density of IIE in canonical (red) and 3′ non-canonical (pale red) splice sites. The positional density is smoothed over a window of 10 bases.

In order to analyze how these different SRE patterns influence exon/intron recognition, we classified the ESE/ISS sequences as exon-identity elements (EIEs) and ISE/ESS sequences as intron-identity elements (IIEs) (30), and calculated their positional density within 5′/3′ exonic and intronic windows 100 nt long (Figure 4). As all of U2/U12-like non-canonical splice sites have only one non-canonical dinucleotide, we classified them into 5′ and 3′ non-canonical splice sites. Comparing EIE/IIE densities in exon–intron boundaries of canonical and non-canonical splice sites, we found that all significant differences indicate an overall enrichment of SREs along exon–intron boundaries that have non-canonical splice sites (Figure 4 and Supplementary Table S2). The same significant differences were observed when EIE/IIE densities in exon–intron boundaries of non-canonical splice sites were compared with EIE/IIE densities from alternative canonical splice sites (Supplementary Table S3).

### Some non-canonical splice sites are modified by RNA editing

**Figure 5.**
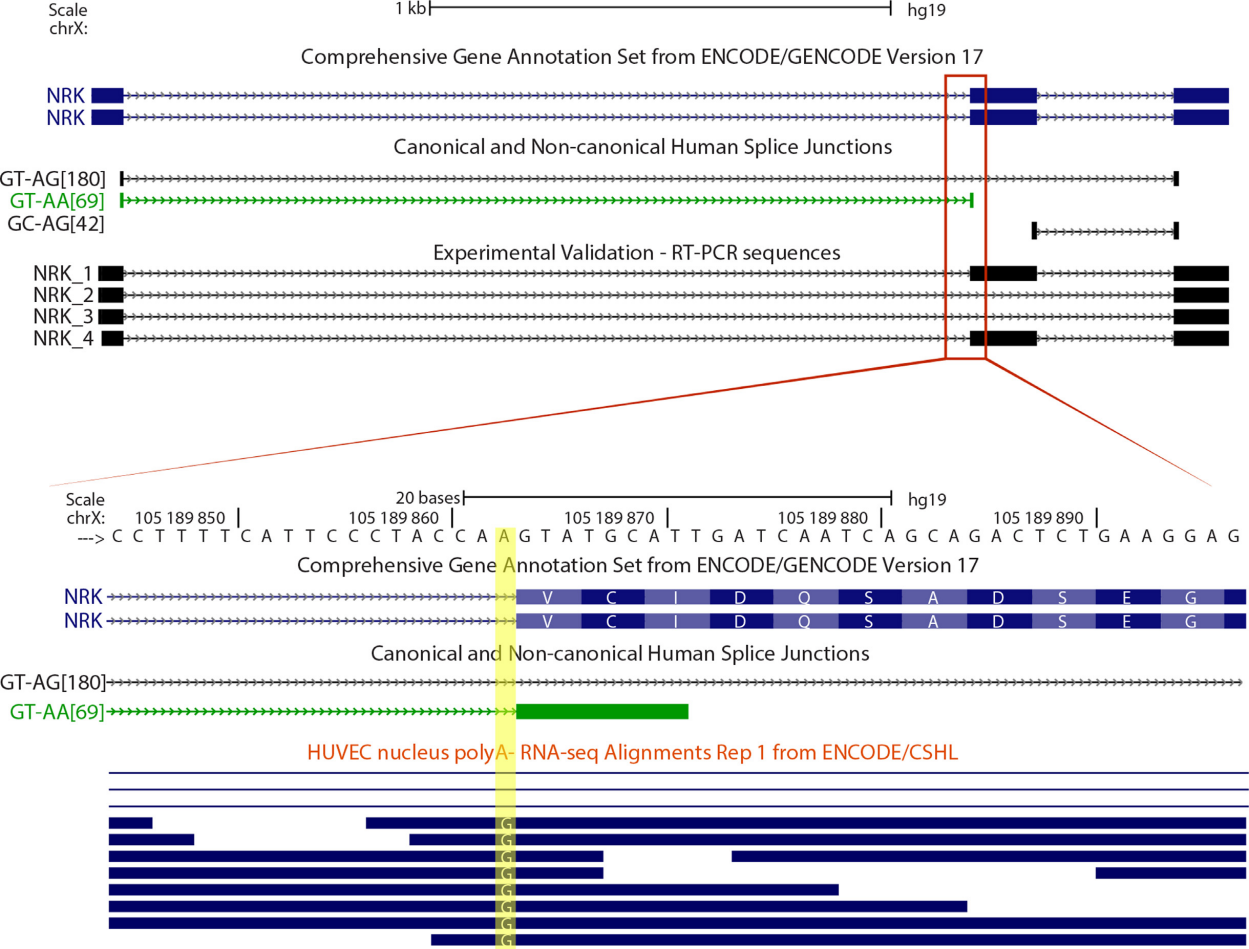
Editing-dependent splicing of non-canonical splice sites. The adenine of the AA-3′ non-canonical splice site that is highlighted in yellow shows a consistent A>G mismatch in poly(A)-minus RNA-seq data from HUVEC cell line. This reflects an A-to-I editing event in the AA-3′ non-canonical splice site, which likely allows the splicing of the GT–AA intron and the exon inclusion of a cassette exon. The exon skipping event is not annotated in GENCODE v17.

Alternative splicing could be influenced by RNA editing. In particular, A-to-I editing was found to directly modify three reported cases of non-canonical splice sites (41–43). Because inosine is recognized as guanine by the spliceosome, alternative 5′-AT or AA-3′ can be turned into canonical splice sites through A-to-I editing. Since inosine is base-paired with cytosine during reverse transcription, sequencing machines also identify inosine as guanosine. We found three AT–AG and four GT–AA non-canonical splice sites that have consistent A>G mismatches in poly(A)-minus RNA-seq alignments from ENCODE cell lines (Table 2). These splice sites are involved in alternative splicing. For example, when the GT–AA non-canonical splice site of NRK gene is edited to GT–AI, a 42 nucleotide exon is included, making this an A-to-I editing dependent exon inclusion event (Figure 5).

**Table 2.** Edited U2/U12-like non-canonical splice sites

Gene	Intron type	Edited dinucleotides	Reference
NRK	U2-like	GT–A**A** > GT–A**G**	
ADARB1	U2-like	GT–A**A** > GT–A**G**	(41)
HOOK3	U2-like	GT–A**A** > GT–A**G**	
COG8	U2-like	GT–A**A** > GT–A**G**	
C14orf37	U2-like	**A**T–AG > **G**T–AG	
NUP210	U12-like	**A**T–AG > **G**T–AG	
CRYZL1	U12-like	**A**T–AG > **G**T–AG	

### Non-canonical introns as a source of transcript annotation errors

**Figure 6.**
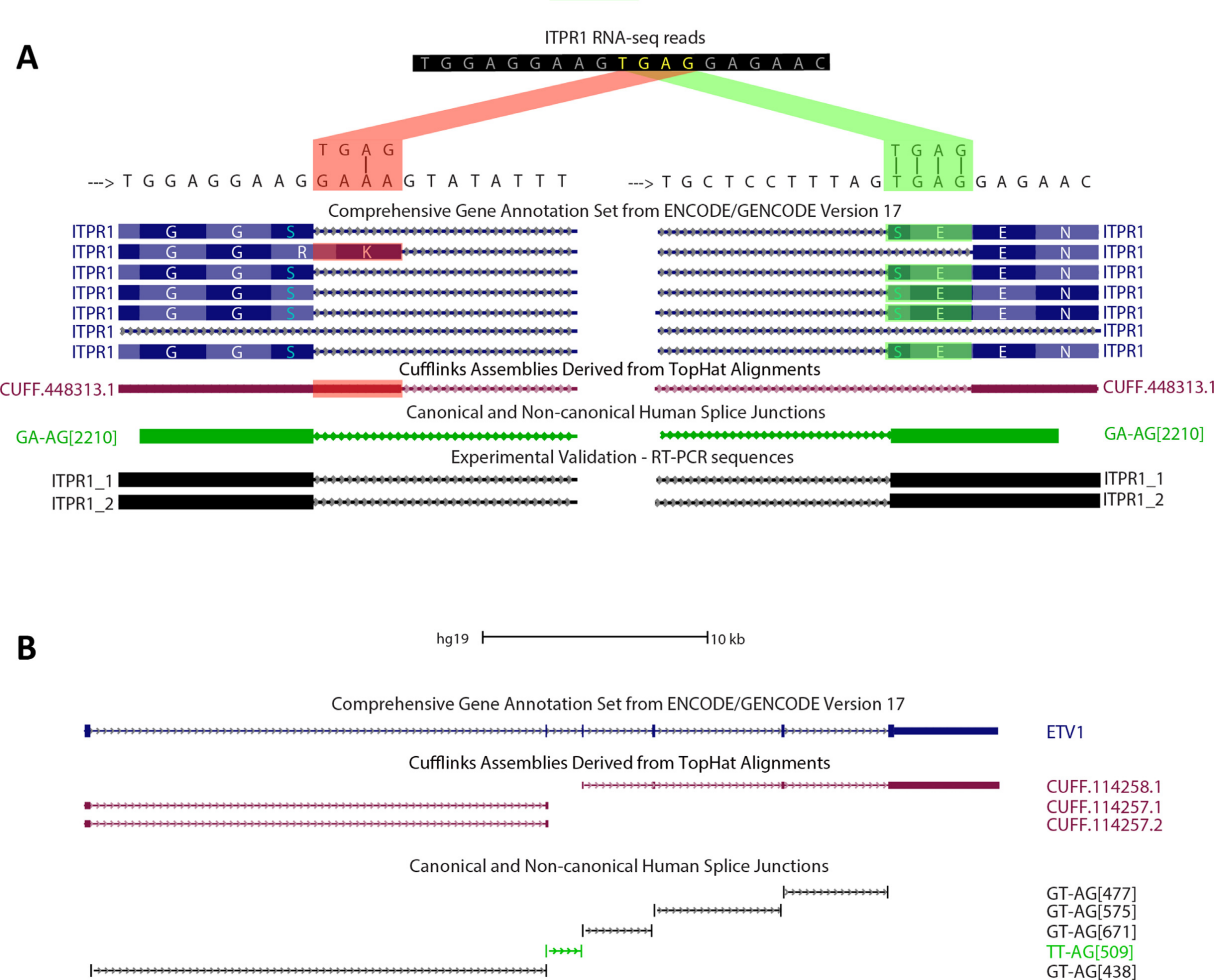
Non-canonical splice sites are prone to be misannotated. (**A**) UCSC Genome Browser image shows a splice site area of ITPR1 where RNA-seq reads can be aligned in two ways. The difference between the two alignments relies on a TGAG sequence (yellow letters) that can be aligned with three mismatches, evidencing a canonical GT–AG splice junction (red alignment) or without mismatches, but evidencing a non-canonical GA–AG splice junction (green alignment). A GENCODE isoform is based on the suboptimal alignment (highlighted in red). Assembled transcript of ITPR1 based on Tophat RNA-seq alignments is based on the suboptimal alignment (highlighted in red). RT-PCR coupled to Sanger sequencing probed that this transcript does not have any mismatches with the genome. (**B**) ETV1 gene has a constitutive TT–AG non-canonical splice junction that is annotated in GENCODE, but Cufflinks cannot assemble a continuous transcript for ETV1 due to TopHat's inability to align non-canonical splice junctions.

The existence of non-canonical introns is underestimated by many widely used RNA-seq mappers (44–49). For this reason, non-canonical introns are a frequent source of transcriptome misannotations. Some misannotations are due to the presence of putative GT–AG canonical splice sites near the real non-canonical splice sites. For example, the reads of a GA–AG non-canonical splice junction in the ITPR1 gene can be misaligned (with three mismatches) to a false canonical GT–AG (Figure 6A). This artifact is produced because some RNA-seq mappers, like TopHat (44), cannot align the reads to the non-canonical splice junction even though this alignment does not contain mismatches. Instead of this, TopHat aligns the reads with three mismatches near 5′-GT and AG-3′ splice sites. Moreover, this error is also present in the GENCODE annotation. The alignment of the cDNAs that we obtained by RT-PCR and Sanger sequencing corroborates the mRNA sequence and splicing of the non-canonical GA–AG intron (Figure 6A).

TopHat can only map reads to canonical splice junctions (GT–AG, GC–AG and AT–AC) (44–49). For this reason, when there are no putative canonical splice sites near to actual non-canonical splice junctions, TopHat is unable to align the RNA-seq reads. This is observed at ETV1 transcripts, where TopHat cannot align RNA-seq reads to a TT–AG non-canonical splice junction, so the reads cannot be assembled into one single transcript by Cufflinks (50) (Figure 6B).

### The majority of non-canonical splice sites that do not have U2/U12-like sequences may be artifacts

The intron with non-canonical splice sites of XBP1 is the only mRNA intron that is known to be processed by non-spliceosome machinery (51), so it does not have U2/U12-like sequences. In the present analysis, we found 278 introns without U2/U12-like sequences around their splice sites (non-U2/U12) (Figure 1C). The fact that we detected the known non-canonical splice sites of XBP1 gene indicates that the methodology used for this work is sensitive to find splice junctions that are not processed by the spliceosome (Supplementary Figure S10).

Among the non-U2/U12 splice sites, we found 83 predicted splice sites that have their 5′ and 3′ ends near (≤10 nt) to a canonical splice junction (Supplementary Data). However, 65 of these predicted non-canonical splice sites are flanked by 5′ or 3′ exonic homopolymers of 5 nt or longer. The detection of these false non-canonical introns can be explained by misalignments to canonical splice junctions due to homopolymer associated indels.

**Figure 7.**
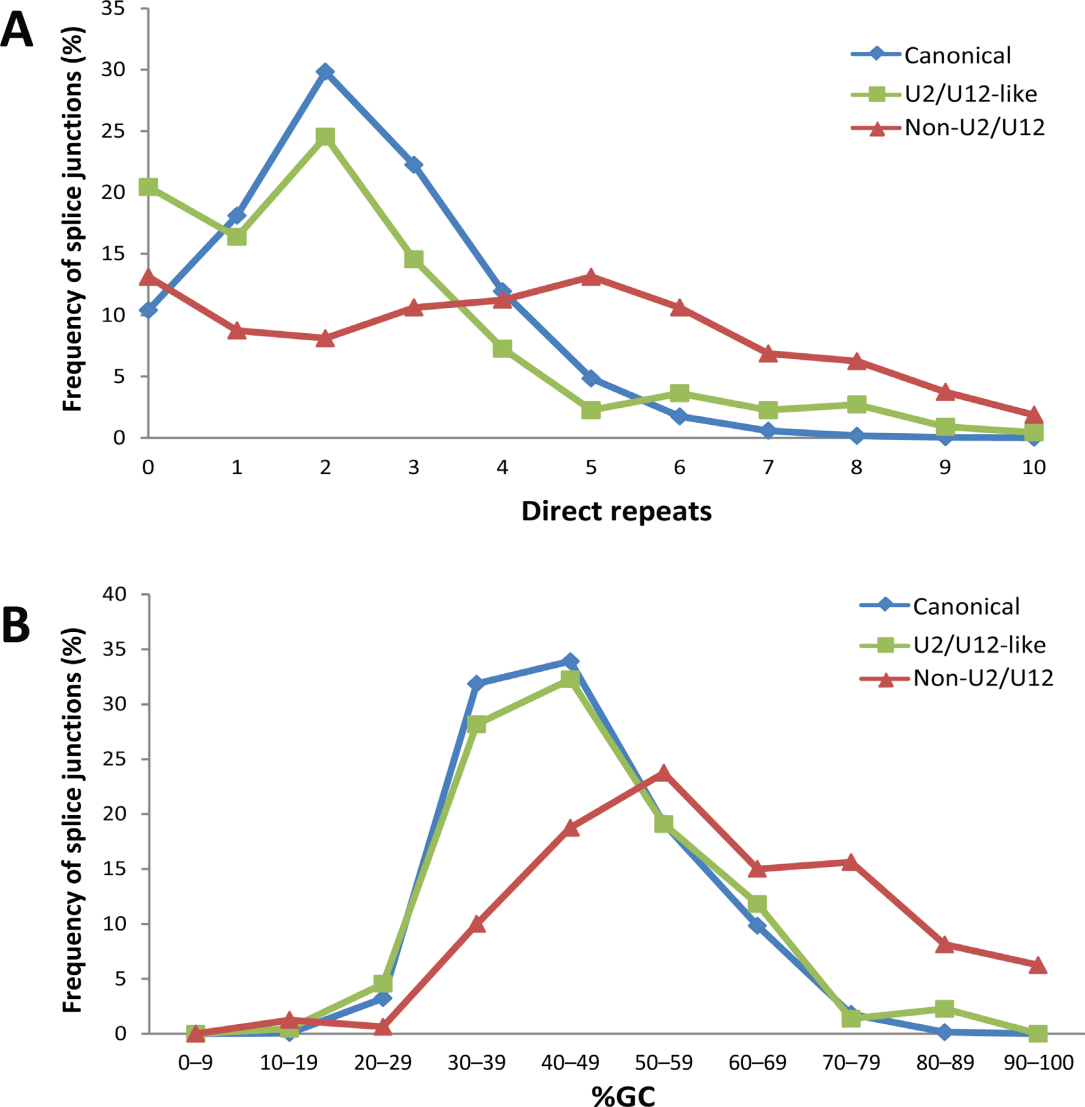
Non-U2/U12 splice junctions have a higher number of direct repeats and %GC content. (**A**) Frequency distribution of the number of direct repeats at canonical, U2/U12-like non-canonical and non-U2/U12 non-canonical splice junctions. (**B**) Frequency distribution of %GC content in canonical, U2/U12-like non-canonical and non-U2/U12 splice junctions.

**Figure 8.**
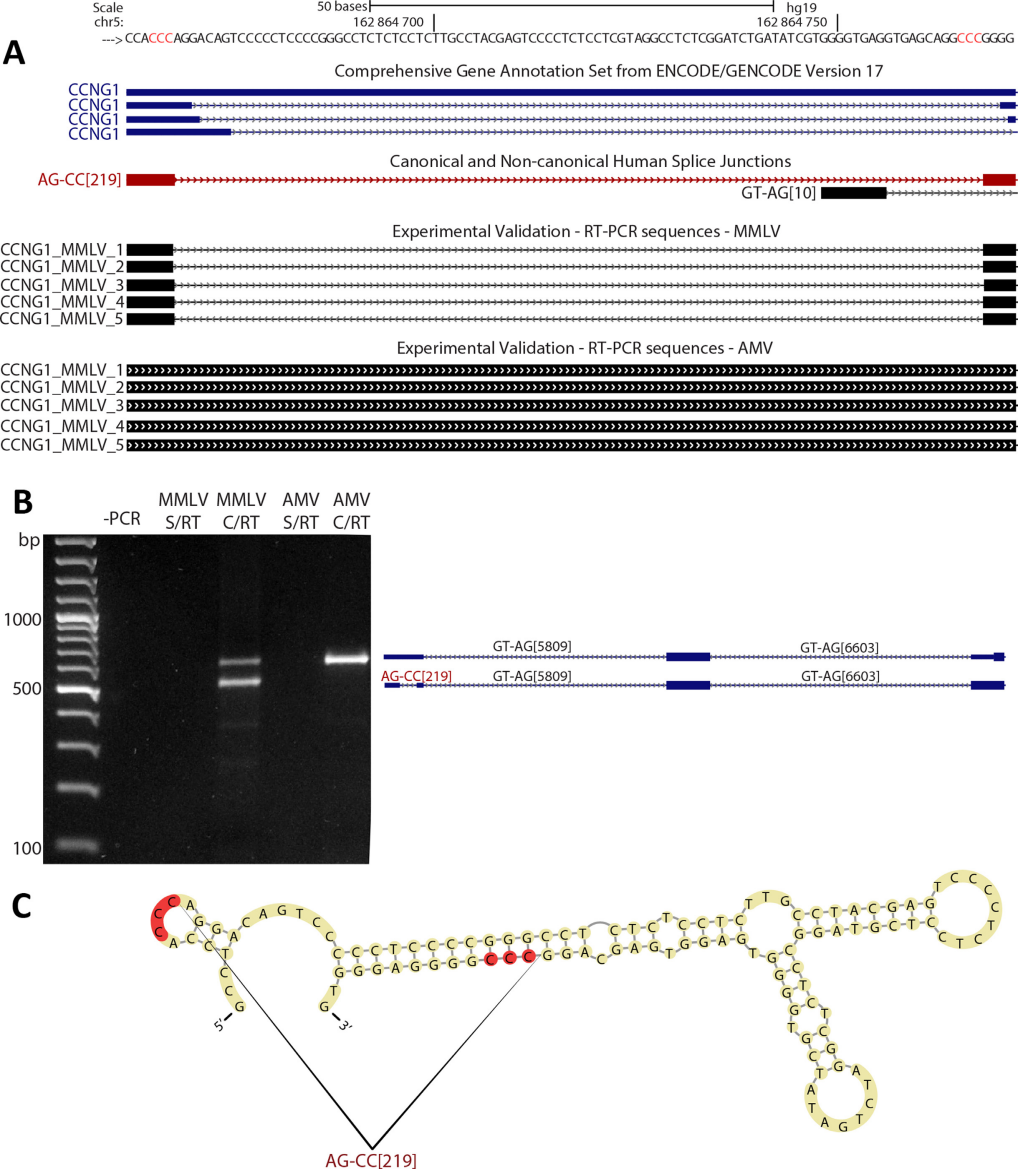
The non-U2/U12 splice junction of CCNG1 is a template switching artifact. (**A**) Our human splice junction annotation shows a non-U2/U12 splice junction (AG–CC[219]) present in the 5′ UTR of CCNG1 gene. Other non-U2/U12 splice junctions for this gene are annotated in GENCODE, but only the non-U2/U12 splice junction from our annotation (shown in red) was obtained by RT-PCR and Sanger sequencing. Red genomic letters indicate the 3-nt long direct repeat associated with this splice junction. (**B**) RT-PCR of CCNG1 transcripts using MMVL or AMV enzymes; alongside are represented the different products amplified. (**C**) *In silico* prediction of the secondary RNA structure associated with the CCNG1 non-U2/U12 splice junction. Directs repeats are highlighted in red.

Template switching during reverse transcription is also a potential source of false non-canonical splice junctions (52–55). When a RNA template contains direct repeats, RT can switch from one portion of the template to another generating a deletion in the cDNA. This phenomenon could generate false splicing-like events during cDNA library construction and it has been shown to be favored by the presence of direct repeats and secondary structures (52–55). We found 160 predicted non-canonical splice junctions that do not have U2/U12-like sequences and are not near to canonical splice sites (Supplementary Data). These predicted non-canonical splice junctions show longer direct repeats and higher %GC than canonical and non-canonical U2/U12-like splice junctions (Figure 7). Taken together, the data suggest that this group of predicted non-canonical splice junctions is highly enriched in template switching artifacts. We tested the veracity of the predicted non-U2/U12 splice junctions present in CCNG1, PSENEN and NAPA genes through RT-PCR and Sanger sequencing. The RT-PCR products that evidence the predicted non-U2/U12 splice junctions were obtained in highly variable proportion depending on which RT enzyme was used (Figure 8, Supplementary Figures S11 and S12). For example, we tested the reproducibility of the detection of CCNG1 non-U2/U12 splice junction by RT-PCR using MMLV or AMV RT enzymes. The CCNG1 non-U2/U12 splice junction was detected in MMLV RT-PCR products, but was not observed when AMV was used (Figure 8A and B). Similar results were obtained with the PSENEN and NAPA RT-PCRs (Supplementary Figures S11 and S12). Moreover, these predicted non-U2/U12 splice junctions are associated with high %GC RNA secondary structures (Figure 8C, Supplementary Figures S11C and S12C).

## DISCUSSION

We have done a comprehensive analysis of human non-canonical splice sites based on deep transcriptome sequencing data generated by RNA-seq. Our method was developed to avoid false non-canonical splice site sources, like alignment errors and polymorphisms. We found 184 U2/U12-like non-canonical splice sites and 51% of them are not annotated on GENCODE v.17. Our results represent a reliable catalog of non-canonical splice sites.

At least 28% of human U2/U12-like non-canonical splice sites are conserved in the mouse transcriptome. Even more, we found examples of U2/U12-like non-canonical splice sites that are highly conserved across vertebrates. The evolutionary conservation supports the idea that this group of splice sites is neither experimental artifact nor spliceosomal errors. Conversely, evolutionary conservation suggests a putative role of non-canonical splice sites in gene regulation. Furthermore, the human U2/U12-like non-canonical splice sites that are not conserved in mouse transcriptome may come from recent evolutionary events as the primate-specific non-canonical splice site that we found in ACTR10 (Figure 2B).

Our analysis shows that U2/U12-like non-canonical splice sites are highly involved in alternative splicing in comparison with canonical splice sites. Alternative splice sites are reported to be weaker than the constitutively processed splice sites and this weakening of splice sites has been proposed as a mechanism of alternative splicing through evolution (56,57). Thus, the weak splice signals of the U2/U12-like non-canonical splice sites could lead to alternative splice site activation. This is reflected on a splice junction in BANP gene in which only the vertebrates that have non-canonical splice sites have evidences of alternative 3′ splice site usage (Figure 2A).

Our data show that several alternative splicing events associated with U2/U12-like non-canonical splice sites are tissue-specific. For instance, the CPSF3 non-canonical splice junction was only detected in testes. Others, like TSPYL2 non-canonical splice junction, show different levels of selection across human tissues. Moreover, the use of TSPYL2 non-canonical splice site generates a premature termination codon that disrupts a functional NAP-like motive and may induce non-sense mediated decay of TSPYL2 transcripts. This could be a regulatory mechanism of tissue-specific TSPYL2 expression, as has been shown for other mammalian genes (58–60).

SREs can recruit *trans*-acting splicing factors that modulate alternative splicing (61). We found an enrichment of SREs nearby non-canonical dinucleotides, the highest increase found in 5′ regions of 5′ non-canonical splice sites and 3′ regions of 3′ non-canonical splice sites. The SREs are shown to have a key role for alternative 5′/3′ selection (62–64), which are the most frequent type of alternative splicing events involving non-canonical splice sites. The weak splice signals of non-canonical splice sites and the high SRE density at their proximal regions indicate a high regulatory potential in the selection of these splice sites. In addition, this may explain the high percentage of non-canonical splice sites that are involved in alternative splicing events.

Most of the U2/U12-like splice sites have dinucleotides with only one mismatch with the canonical splice site dinucleotides. However, a single base mismatch at canonical U2 splice site dinucleotides induces dramatic functional impairments (3) and is frequently associated with mis-splicing diseases (65–69). Why these splice junctions can be processed even if they have non-canonical splice sites? It has been reported that two GT–AA and one AT–AG splice sites are converted into canonical splice sites by RNA A-to-I editing (41–43). We found five GT–AA and three AT–AG non-canonical splice sites that show evidence to be edited at their dinucleotides, and six of them are novel examples of this phenomenon (Table 2). Moreover, this RNA A-to-I editing enhances the selection of the edited non-canonical splice sites (Figure 5). However, we also found GT–AA and AT–AG non-canonical splice sites that do not show evidence of RNA A-to-I editing. The splicing of the remaining U2/U12-like splice sites cannot be explained by this mechanism.

Previous investigations have shown that U12 introns can be processed correctly by using combinations of terminal dinucleotides different from the known AT–AC and GT–AG. Analysis of splice sites in databases from different organisms (6) and point mutational analysis of U12-dependent splice sites (4) showed that a 5 ′A can splice to any 3′ nucleotide (AT–AN) although with a preference for 3′ AC dinucleotide. A 5 ′G can splice to 3 ′G or T nucleotide (GT–AG or GT–AT) with a preference for 3′ G nucleotide. This is consistent with our data as shown in Table 1. U12 spliceosome has a different mechanism of recognizing splice sites. It has been shown that the U11 and U12 snRNAs associate with each other before binding to the 5′ss and branch points, respectively (70). Since the U12 branch point plays a much bigger role in splice site recognition, this leads to a more relaxed constraint on the 3′ splice site dinucleotides. Burset *et al.* (13) proposed that non-canonical splice sites could function exclusively in association with near canonical splice sites that can efficiently recruit the splicing machinery, presenting a parasitic non-canonical/canonical splice site relationship. This mechanism has been shown for the GA–AG non-canonical splice sites present on FGF1, where the 5′-GA non-canonical splice site recognition depends on 5′-GT canonical splice site situated 6 nt upstream and two intronic sequences downstream (9). However, more evidence is needed to know if all non-canonical splice sites depend on canonical splice sites to be recognized. Moreover, this mechanism cannot explain the constitutive non-canonical splice site processing that represents 24% of the non-canonical splice sites found by our analysis. Thus, more experimental evidence is necessary to understand the molecular mechanisms that contribute to non-canonical splice site selection.

Detection of non-canonical splice sites is often attributed to artifactual events (13,71–73). However, real non-canonical splice sites are frequently misannotated and underestimated by most used RNA-seq analysis tools (44–49). The presence of non-canonical splice sites at human transcripts generates false canonical splice site detection or disruption of assembled transcripts, evidencing that transcript annotation pipelines and RNA-seq tools have a deficient methodology to do *ab initio* detection of non-canonical splice sites. Thus, our annotation of non-canonical splice sites will lead to an improvement of the human transcriptome annotation.

In the present analysis, extensive methods were designed to avoid false non-canonical splice site detection. However, the non-U2/U12 splice sites represent a group of splice sites that could be highly affected by sequencing errors and reverse transcription artifacts during cDNA library construction. The Illumina sequencing platform, used to generate the RNA-seq data, is considered robust against indel errors; however, within homopolymers, the indel error rate is dramatically higher (74). We found 64 non-U2/U12 splice sites that are flanked by 5′ or 3′ exonic homopolymers. These non-U2/U12 splice sites are ≤10 nt from canonical splice sites. Thus, non-U2/U12 splice sites are more likely to be homopolymer-associated indels than U2/U12-like splice sites.

False non-canonical splice sites can be generated by RT template switching, a phenomenon that is enhanced by secondary RNA structures and the presence of direct repeats at the splice junctions (52–55). The group of non-U2/U12 splice sites identified herein has longer direct repeats and higher %GC than canonical and non-canonical U2/U12-like introns, suggesting an enrichment of reverse transcription artifact in non-U2/U12 introns. RT-PCRs designed to amplify non-U2/U12 splice junctions present on CCNG1, PSENEN and NAPA genes show different results depending on the RTs used. These splice junctions are associated with high %GC introns and RNA secondary structures, suggesting that at least these three non-canonical splice sites are artifacts. These results indicate that a RT-independent sequencing method of RNA is needed to make a confident catalog of non-U2/U12 splices sites.

In conclusion, here we report the landscape of non-canonical splice sites present at the human transcriptome. We identified a high confidence group of 184 U2/U12-like non-canonical splice sites that will improve the human transcriptome annotation. Non-canonical splice site selection is a rare event in human transcriptome associated with distinctive features. Almost all U2/U12-like non-canonical splice sites have only one non-canonical dinucleotide, which is often associated with alternative canonical dinucleotide. Additionally, the high SRE density of exon–intron boundaries that have U2/U12-like non-canonical splice sites gives an appropriated context to process these weak splice sites in a regulated fashion.

## SUPPLEMENTARY DATA

Supplementary Data are available at NAR Online.

SUPPLEMENTARY DATA
