# Supplementary material for: A comprehensive survey of non-canonical splice sites in the human transcriptome
Source: Nucleic Acids Res. 2014 Aug 14;42(16):10564–78. doi: 10.1093/nar/gku744 (PMC4176328; doi:10.1093/nar/gku744)
Supplement: SUPPLEMENTARY DATA [file supp_42_16_10564__index.html]

A comprehensive survey of non-canonical splice sites in the human transcriptome — SUPPLEMENTARY DATA 

# A comprehensive survey of non-canonical splice sites in the human transcriptome

## SUPPLEMENTARY DATA

**Files in this Data Supplement:**

- SUPPLEMENTARY DATA
- SUPPLEMENTARY DATA
