# Supplementary material for: A comprehensive survey of non-canonical splice sites in the human transcriptome
Source: Nucleic Acids Res. 2014 Aug 14;42(16):10564–78. doi: 10.1093/nar/gku744 (PMC4176328; doi:10.1093/nar/gku744)
Supplement: SUPPLEMENTARY DATA [file supp_gku744_SUPPLEMENTARY_TABLE_AND_FIGURES_LEGENDS_2.pdf]

SUPPLEMENTARY TABLE AND FIGURES LEGENDS

Figure S1-9. U2/U12-like Non-canonical splice junctions present at ETV1, IDH3B, ITPR1, NRK, C1orf63, POLR2E, RBFOX2 and TSPYL2 were validated by RT-PCR and Sanger sequencing. However, the U2/U12-like non-canonical splice junction present at CTNNB1 was not detected in the sequenced subclones. A) UCSC genome browser images show the identified non-canonical U2/U12-like splice junctions and the Sanger sequences that confirm their existence. B) RT-PCR of the non-canonical U2/U12-like splice junctions using 3 different RTs. The correspondence of RT-PCR products and expected isoforms are show to the right of each gel image, where § indicate that the isoform is not annotated in GENCODE v.17 and * indicate that the presence of the isoform was corroborated by Sanger sequencing.

Table S1. The primers used for RT-PCRs of the U2/U12-like and non-U2/U12 non-canonical splice junctions.

Table S2. Odd ratios and Pearson Chi-square P-values calculated of the differences of IE densities between the different regions of U2/U12-like non-canonical and canonical splice sites.

Table S3. Odd ratios and Pearson Chi-square P-values calculated of the differences of IE densities between the different regions of U2/U12-like non-canonical and alternative canonical splice sites.

Figure S10. A picture of UCSC genome browser shows the detection of the non-U2/U12 splice junction of XBP1 gene. Red letters highlight the 6-nt direct repeat associated to this splice junction. As non-canonical splice sites present in GENCODE and in our annotation are located in direct repeats, the two alignments are equivalent.

Figure S11-12. Non-U2/U12 splice junctions present at PSENEN and NAPA were tried to validate through RT-PCR and Sanger sequencing. A) Pictures of UCSC genome browser show the non-U2/U12 splice sites and the alignment of the Sanger sequences. B) RT-PCR of the non-U2/U12 splice junctions using 2 different RTs. C) Secondary RNA structures associated to the non-U2/12 splice junctions. Direct repeats are highlighted in red and orange.

Figure S1

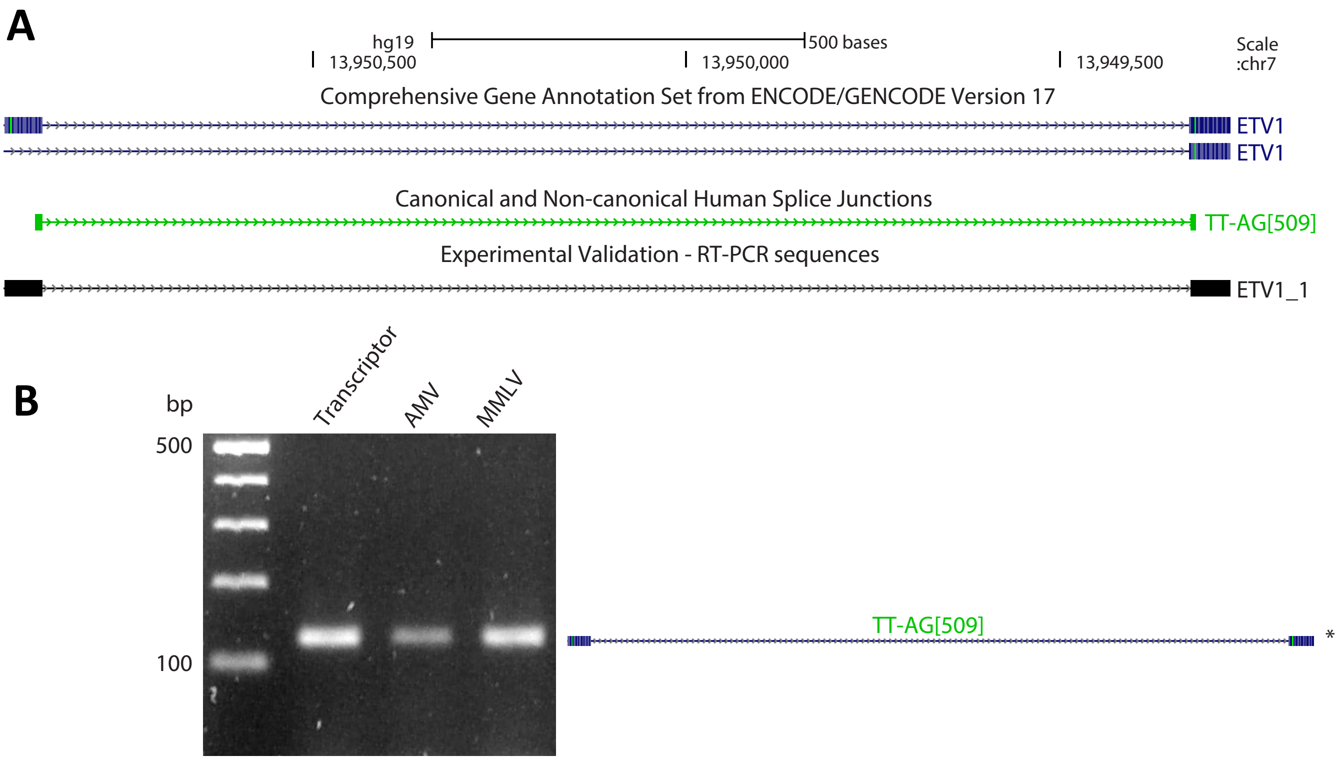

A
hg19
500 bases
Scale
:chr7
13,950,500
13,950,000
13,949,500
Comprehensive Gene Annotation Set from ENCODE/GENCODE Version 17
ETV1
ETV1
Canonical and Non-canonical Human Splice Junctions
TT-AG[509]
Experimental Validation - RT-PCR sequences
ETV1_1
B
bp
Transcriptor
AMV
MMLV
500
100
TT-AG[509]
*

Figure S2

A

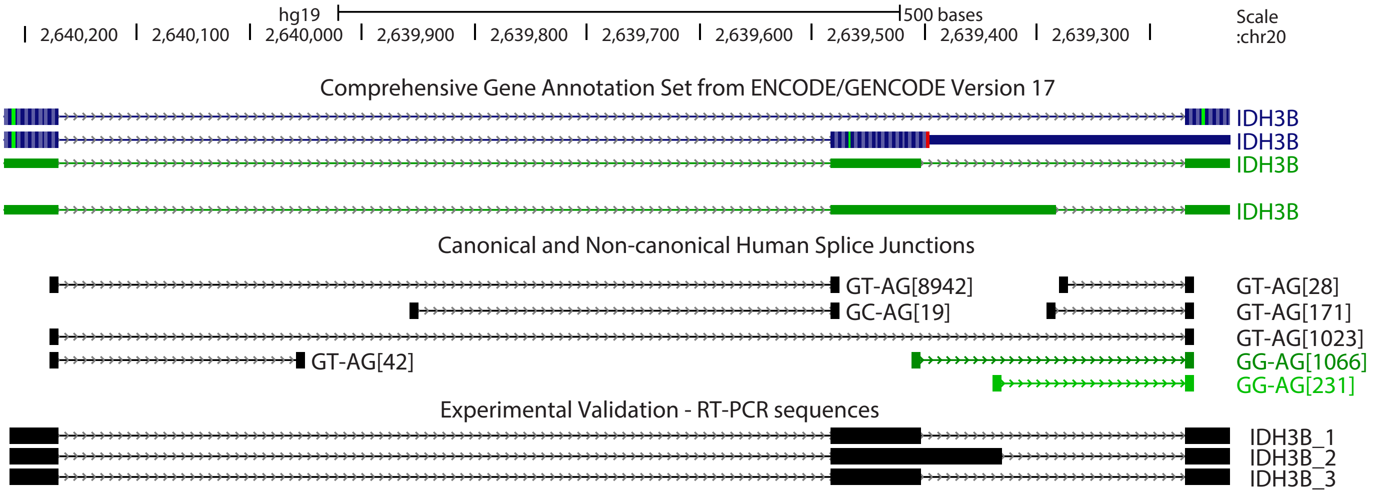

hg19
500 bases
Scale
:chr20
2,640,200
2,640,100
2,640,000
2,639,900
2,639,800
2,639,700
2,639,600
2,639,500
2,639,400
2,639,300
Comprehensive Gene Annotation Set from ENCODE/GENCODE Version 17
IDH3B
IDH3B
IDH3B
IDH3B
Canonical and Non-canonical Human Splice Junctions
GT-AG[8942]
GC-AG[19]
GT-AG[42]
GT-AG[28]
GT-AG[171]
GT-AG[1023]
GG-AG[1066]
GG-AG[231]
Experimental Validation - RT-PCR sequences
IDH3B_1
IDH3B_2
IDH3B_3

B

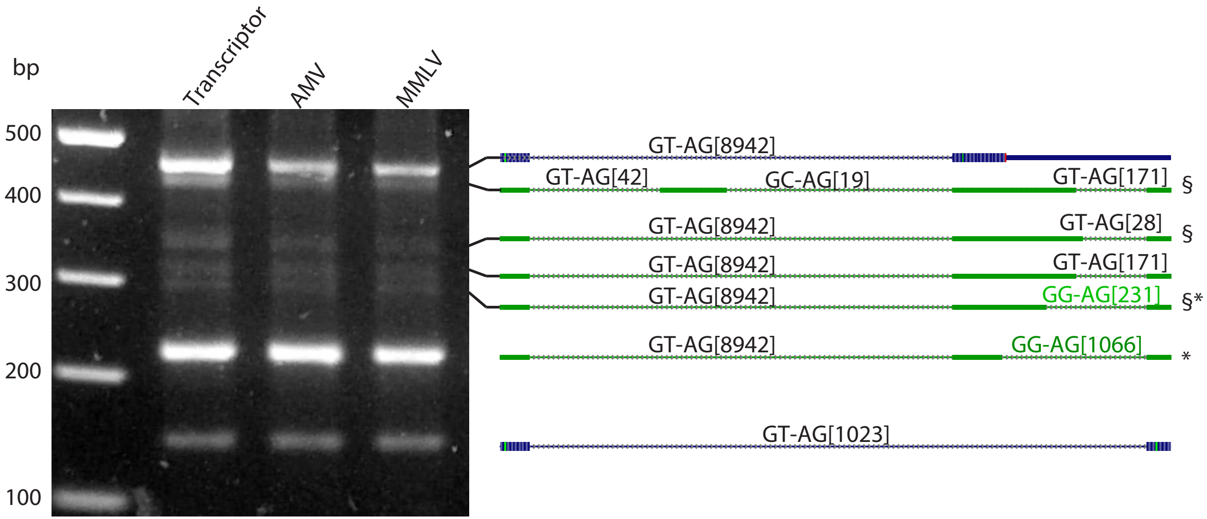

bp
Transcriptor
AMV
MMLV
500
400
300
200
100
GT-AG[8942]
GT-AG[42]
GC-AG[19]
GT-AG[171]
§
GT-AG[8942]
GT-AG[28]
§
GT-AG[8942]
GT-AG[171]
GT-AG[8942]
GG-AG[231]
§*
GT-AG[8942]
GG-AG[1066]
*
GT-AG[1023]

Figure S3

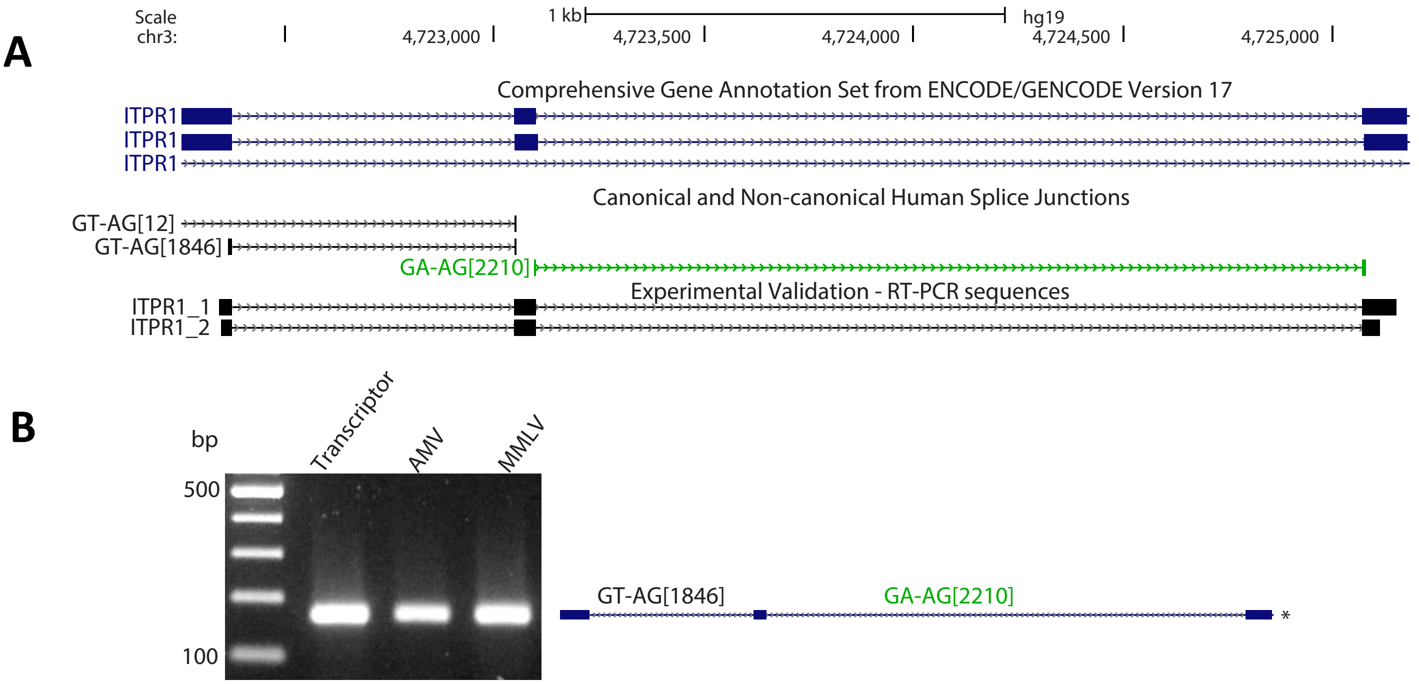
A
Scale
chr3:
1 kb
hg19
4,723,000
4,723,500
4,724,000
4,724,500
4,725,000
Comprehensive Gene Annotation Set from ENCODE/GENCODE Version 17
ITPR1
ITPR1
ITPR1
Canonical and Non-canonical Human Splice Junctions
GT-AG[12]
GT-AG[1846]
GA-AG[2210]
Experimental Validation - RT-PCR sequences
ITPR1_1
ITPR1_2
B
bp
Transcriptor
AMV
MMLV
500
100
GT-AG[1846]
GA-AG[2210]
*

Figure S4

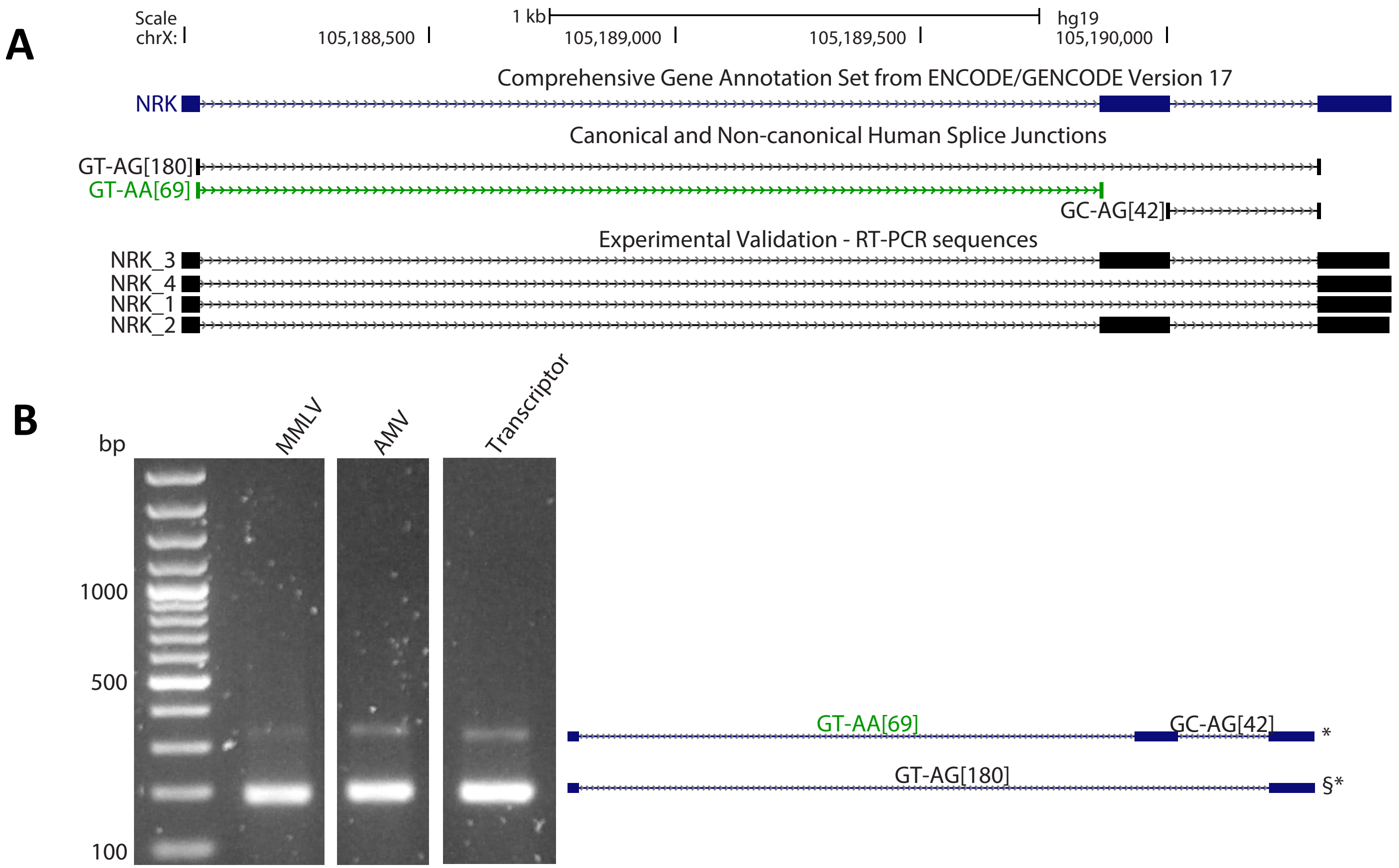
A
Scale
chrX:
1 kb
hg19
105,188,500
105,189,000
105,189,500
105,190,000
Comprehensive Gene Annotation Set from ENCODE/GENCODE Version 17
NRK
Canonical and Non-canonical Human Splice Junctions
GT-AG[180]
GT-AA[69]
GC-AG[42]
Experimental Validation - RT-PCR sequences
NRK_3
NRK_4
NRK_1
NRK_2
B
bp
MMLV
AMV
Transcriptor
1000
500
100
GT-AA[69]
GC-AG[42]
*
GT-AG[180]
§*

Figure S5

A

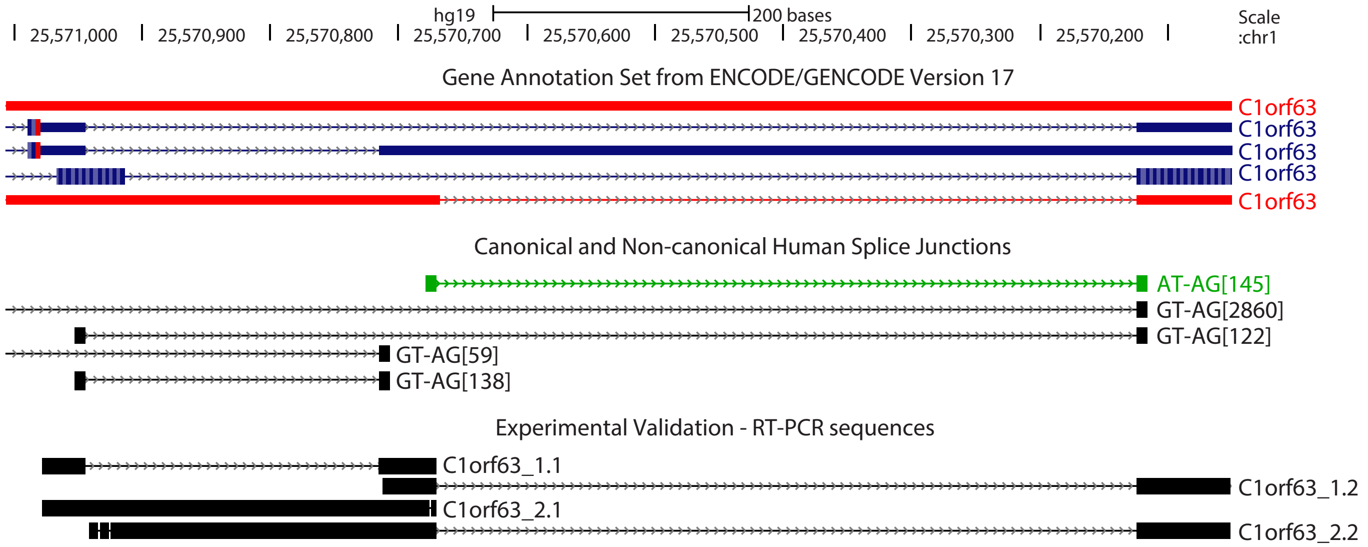
hg19
200 bases
Scale
:chr1
25,571,000
25,570,900
25,570,800
25,570,700
25,570,600
25,570,500
25,570,400
25,570,300
25,570,200
Gene Annotation Set from ENCODE/GENCODE Version 17
C1orf63
C1orf63
C1orf63
C1orf63
C1orf63
Canonical and Non-canonical Human Splice Junctions
AT-AG[145]
GT-AG[2860]
GT-AG[122]
GT-AG[59]
GT-AG[138]
Experimental Validation - RT-PCR sequences
C1orf63_1.1
C1orf63_1.2
C1orf63_2.1
C1orf63_2.2

B

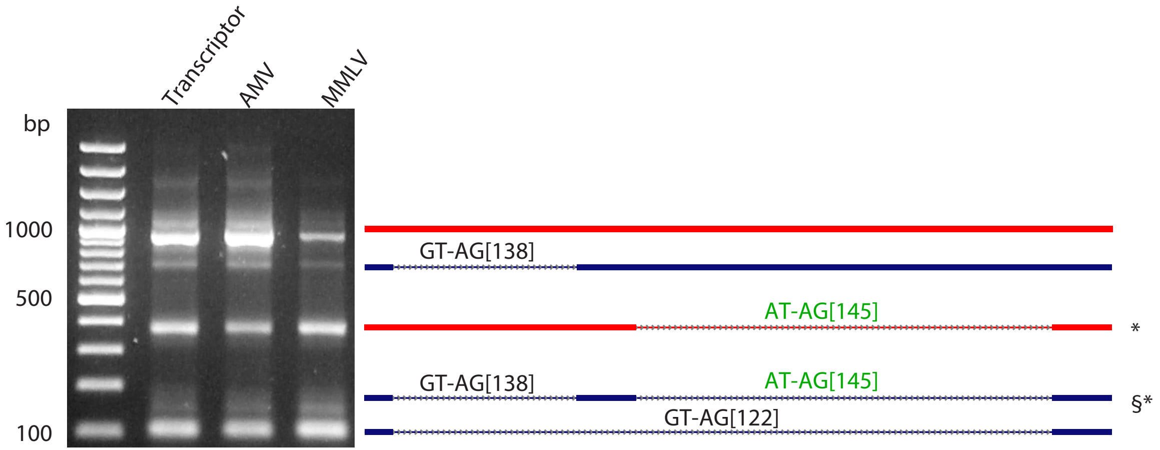
Transcriptor
AMV
MMLV
bp
1000
500
100
GT-AG[138]
AT-AG[145]
*
GT-AG[138]
AT-AG[145]
§*
GT-AG[122]

Figure S6

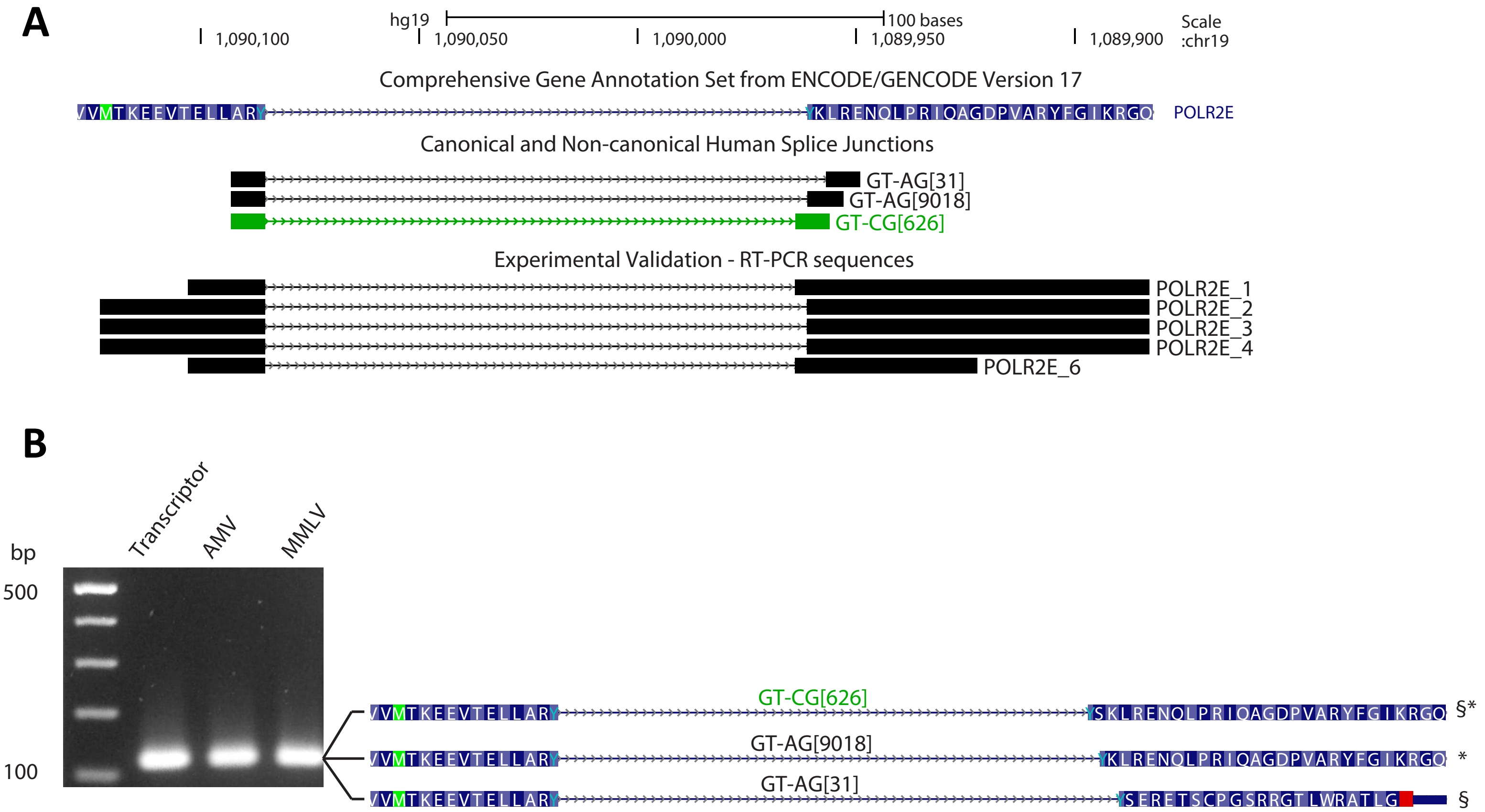
A
hg19
100 bases
Scale
:chr19
1,090,100
1,090,050
1,090,000
1,089,950
1,089,900
Comprehensive Gene Annotation Set from ENCODE/GENCODE Version 17
POLR2E
Canonical and Non-canonical Human Splice Junctions
GT-AG[31]
GT-AG[9018]
GT-CG[626]
Experimental Validation - RT-PCR sequences
POLR2E_1
POLR2E_2
POLR2E_3
POLR2E_4
POLR2E_6
B
bp
Transcriptor
AMV
MMLV
500
100
GT-CG[626]
§*
GT-AG[9018]
*
GT-AG[31]
§

Figure S7

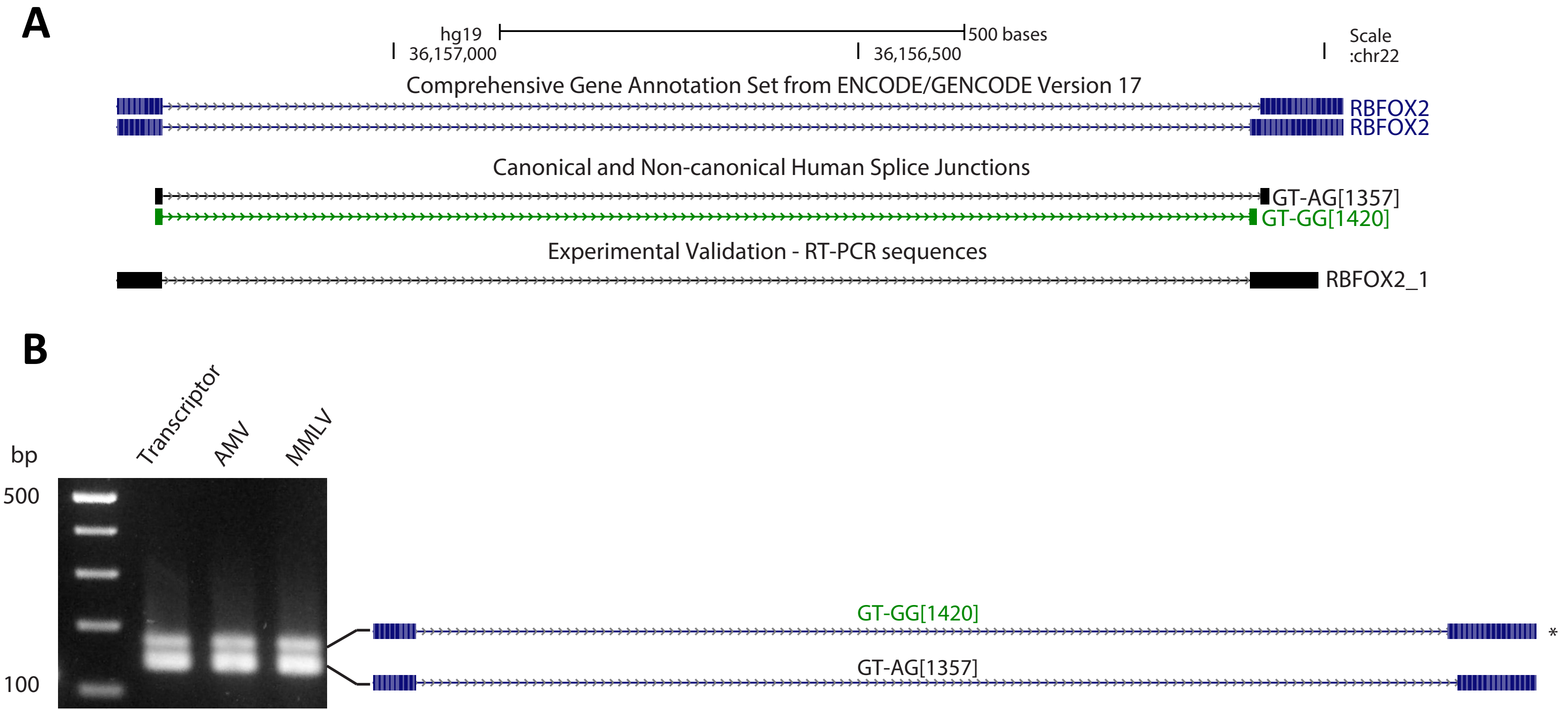

A
hg19
500 bases
36,157,000
36,156,500
Scale
:chr22
Comprehensive Gene Annotation Set from ENCODE/GENCODE Version 17
RBFOX2
RBFOX2
Canonical and Non-canonical Human Splice Junctions
GT-AG[1357]
GT-GG[1420]
Experimental Validation - RT-PCR sequences
RBFOX2_1
B
bp
Transcriptor
AMV
MMLV
500
100
GT-GG[1420]
*
GT-AG[1357]

Figure S8

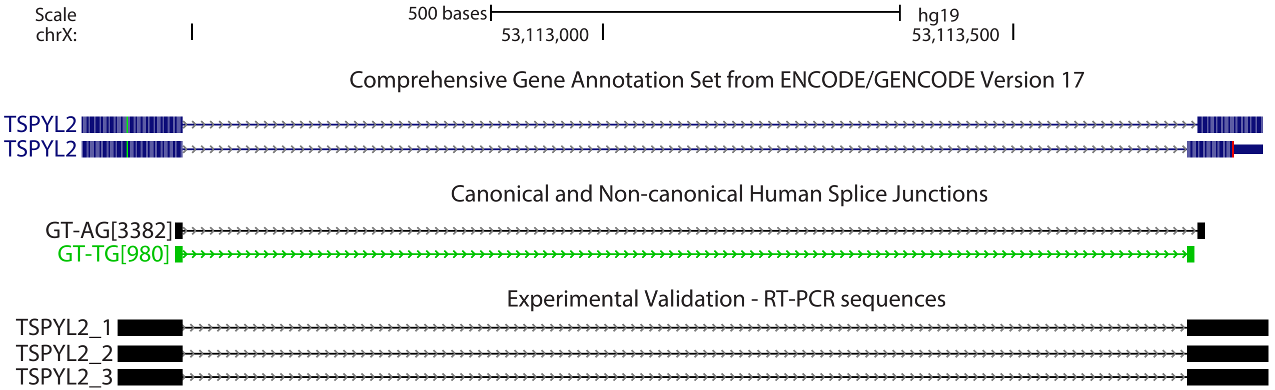

Scale
chrX:
500 bases
hg19
53,113,000
53,113,500
Comprehensive Gene Annotation Set from ENCODE/GENCODE Version 17
TSPYL2
TSPYL2
Canonical and Non-canonical Human Splice Junctions
GT-AG[3382]
GT-TG[980]
Experimental Validation - RT-PCR sequences
TSPYL2_1
TSPYL2_2
TSPYL2_3

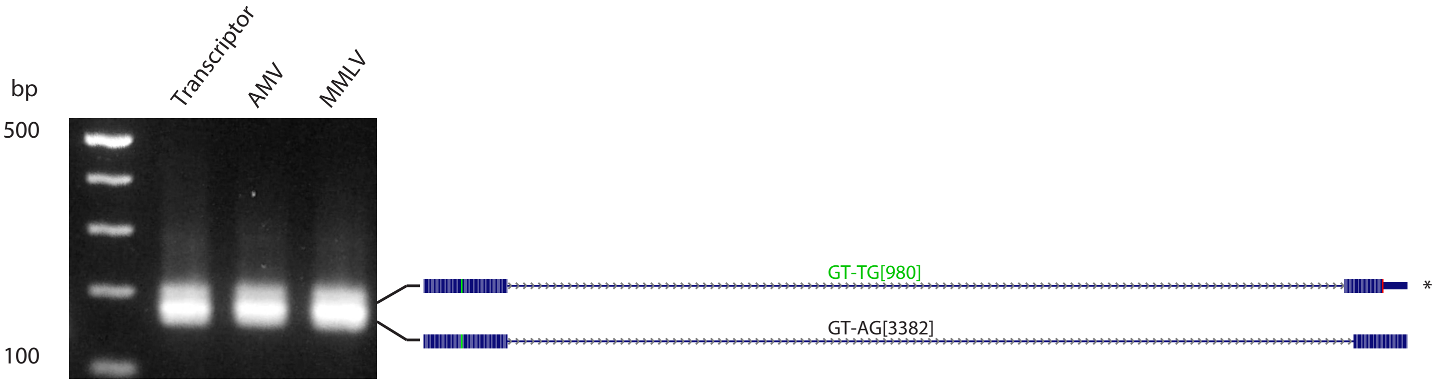

bp
Transcriptor
AMV
MMLV
500
100
GT-TG[980]
*
GT-AG[3382]

Figure S9

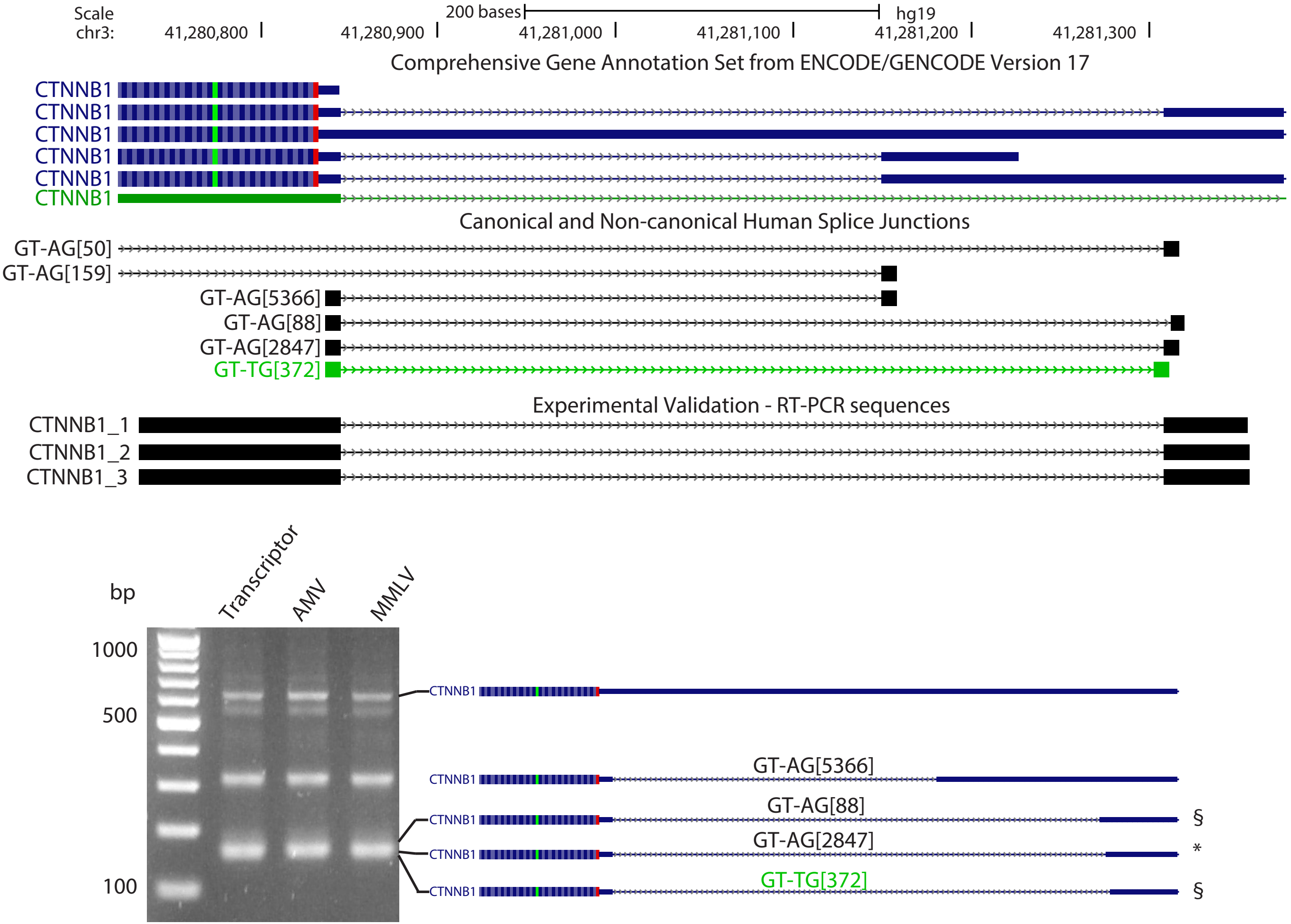
Scale
chr3:
200 bases
hg19
41,280,800
41,280,900
41,281,000
41,281,100
41,281,200
41,281,300
Comprehensive Gene Annotation Set from ENCODE/GENCODE Version 17
CTNNB1
CTNNB1
CTNNB1
CTNNB1
CTNNB1
CTNNB1
Canonical and Non-canonical Human Splice Junctions
GT-AG[50]
GT-AG[159]
GT-AG[5366]
GT-AG[88]
GT-AG[2847]
GT-TG[372]
Experimental Validation - RT-PCR sequences
CTNNB1_1
CTNNB1_2
CTNNB1_3
bp
Transcriptor
AMV
MMLV
1000
500
100
CTNNB1
CTNNB1
GT-AG[5366]
CTNNB1
GT-AG[88]
§
CTNNB1
GT-AG[2847]
*
CTNNB1
GT-TG[372]
§

Table S1

Gene	Primers (5’→3’)
ETVI	AGTGCCTAGCTGCCACTCCATTTA<br>CTGGGACAACACAGGTGTCATCAT
IDH3B	CATGATCGCAGATGCGGTGAAGAA<br>TTACATGGGTATGGACAGGGCCTA
ITPR1	CAGCCAAGAAGGGCCAAGTAATGT<br>AGTCATGCATCGTCAAGTGGAGCA
NKR	CTTTATGCATGGGCACCAAAGTCC<br>TCTTTGGCAGGGTCACATCAGACA
C1orf63	GTGGTCTGCAGACATTGATGAAGAA<br>AGCTATGCTTCTTTGCTGGGTAGG
POLR2E	ATGACCAAGGAGGAGGTGACAGAG<br>TGCCCACGCTTTATCCCAAAGTAG
RBFOX2	ATGTGTCCCTAGGCAATGATGCAG<br>TCAAATGGGCTCCTCTGAAAGCG
CTNNB1	ATATTCCTCGTTAGGGCAGGCG<br>GTCAGTTGCCAATGGGACATTCCT
CCNG1	ATATTCCTCGTTAGGGCAGGCG<br>GTCAGTTGCCAATGGGACATTCCT
PSENEN	GGGTTTGCTTTCCTGCCTTT<br>ATGTCAGCAGAGAACGTGGGACAT
NAPA	ATCAAGAAGACCATCCAGGGCGAT<br>AAGCATTAGGGAAGGAGCAGGTGT

Table S2

U2/U12-like splice sites	Region	Identity elements	Odd ratio	P-value Pearson chi-square
5' non-canonical	5' exon	IIE	1.3855	3.4466E-09
		EIE	1.1474	1.2380E-05
	5' intron	IIE	1.1375	1.6969E-03
		EIE	1.1493	1.4575E-04
	3' intron	IIE	1.0039	9.3516E-01
		EIE	1.1166	3.5408E-03
	3' exon	IIE	1.3856	3.4217E-09
		EIE	1.1102	7.4518E-04
3' non-canonical	5' exon	IIE	0.9406	2.4444E-01
		EIE	1.1047	9.7384E-05
	5' intron	IIE	1.1069	1.5311E-03
		EIE	1.0146	6.2872E-01
	3' intron	IIE	1.1167	1.8233E-03
		EIE	1.1833	3.1760E-09
	3' exon	IIE	0.9406	2.4495E-01
		EIE	1.1191	5.0888E-06

Table S3

U2/U12-like splice sites	Region	Identity elements	Odd ratio	P-value Pearson chi-square
5' non-canonical	5' exon	IIE	1.3187	6.5445E-07
		EIE	1.1781	2.0693E-07
	5' intron	IIE	1.1229	4.7619E-03
		EIE	1.1761	9.5263E-06
	3' intron	IIE	1.0014	9.9628E-01
		EIE	1.1148	4.3347E-03
	3' exon	IIE	1.3189	6.4759E-07
		EIE	1.1400	2.5601E-05
3' non-canonical	5' exon	IIE	0.8952	3.7673E-02
		EIE	1.1342	8.8481E-07
	5' intron	IIE	1.0927	6.0074E-03
		EIE	1.0382	2.1624E-01
	3' intron	IIE	1.1139	2.4714E-03
		EIE	1.1814	4.9915E-09
	3' exon	IIE	0.8953	3.7870E-02
		EIE	1.1491	1.9077E-08

Figure S10

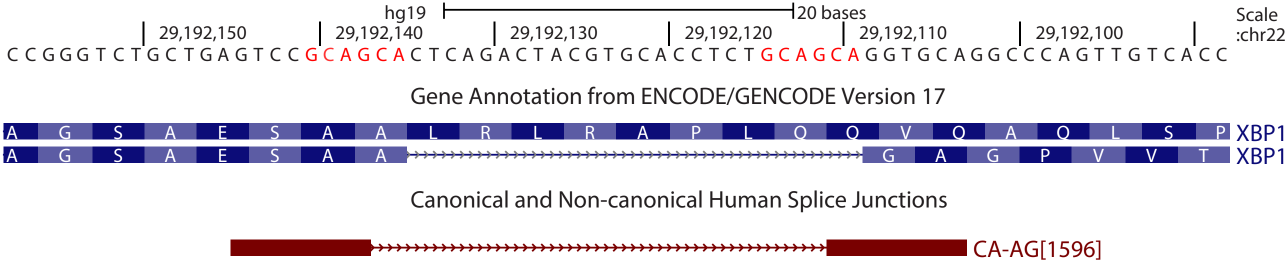
hg19
20 bases
Scale
:chr22
29,192,150
29,192,140
29,192,130
29,192,120
29,192,110
29,192,100
C C G G G T C T G C T G A G T C C G C A G C A C T C A G A C T A C G T G C A C C T C T G C A G C A G G T G C A G G C C C A G T T G T C A C C
Gene Annotation from ENCODE/GENCODE Version 17
A G S A E S A A L R L R A P L Q Q V Q A Q L S P
XBP1
A G S A E S A A G A G P V V T
XBP1
Canonical and Non-canonical Human Splice Junctions
CA-AG[1596]

Figure S11

A

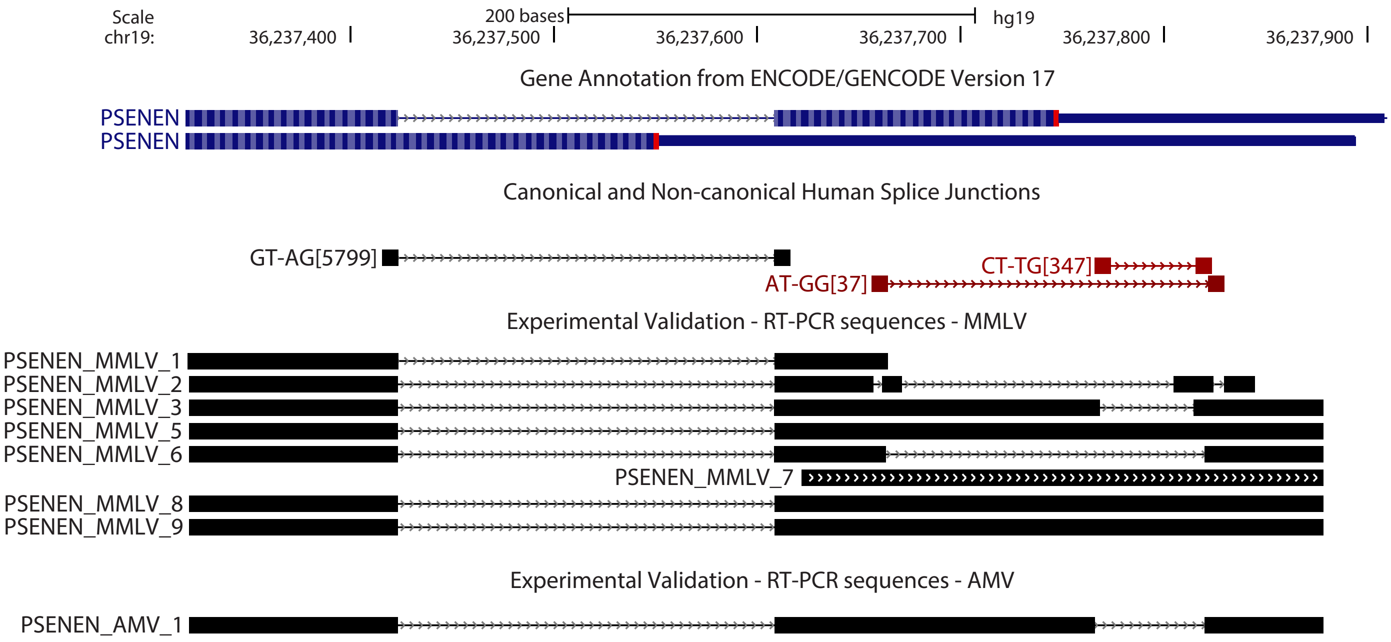

B

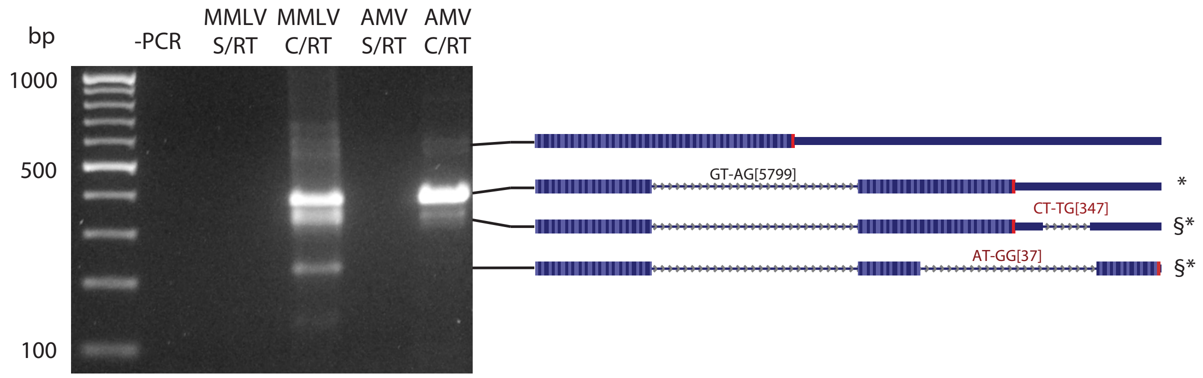

C

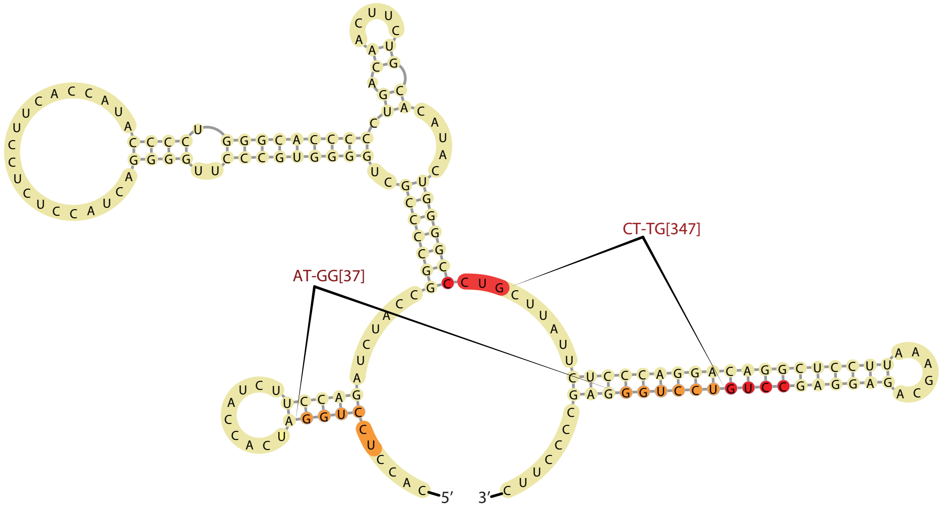

Figure S12

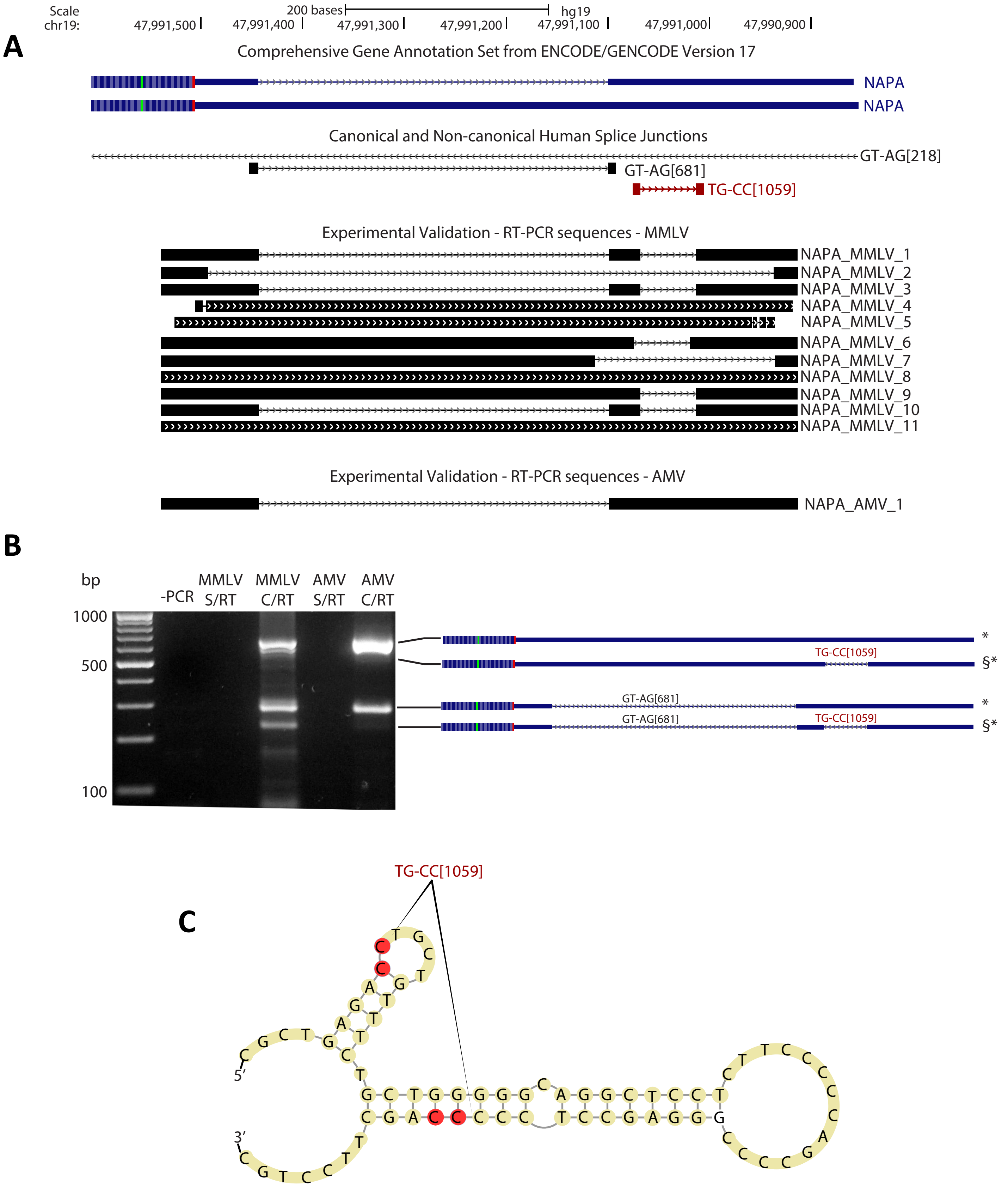
A
Scale
chr19:
200 bases
hg19
47,991,500
47,991,400
47,991,300
47,991,200
47,991,100
47,991,000
47,990,900
Comprehensive Gene Annotation Set from ENCODE/GENCODE Version 17
NAPA
NAPA
Canonical and Non-canonical Human Splice Junctions
GT-AG[218]
GT-AG[681]
TG-CC[1059]
Experimental Validation - RT-PCR sequences - MMLV
NAPA_MMLV_1
NAPA_MMLV_2
NAPA_MMLV_3
NAPA_MMLV_4
NAPA_MMLV_5
NAPA_MMLV_6
NAPA_MMLV_7
NAPA_MMLV_8
NAPA_MMLV_9
NAPA_MMLV_10
NAPA_MMLV_11
Experimental Validation - RT-PCR sequences - AMV
NAPA_AMV_1
B
bp
-PCR
MMLV S/RT
MMLV C/RT
AMV S/RT
AMV C/RT
1000
500
100
*
TG-CC[1059]
§*
GT-AG[681]
*
GT-AG[681]
TG-CC[1059]
§*
C
TG-CC[1059]
5′
3′
